# Construction and validation of a novel prognostic model of neutrophil‑related genes signature of lung adenocarcinoma

**DOI:** 10.1038/s41598-023-45289-8

**Published:** 2023-10-25

**Authors:** Qianjun Zhu, Yanfei Chai, Longyu Jin, Yuchao Ma, Hongwei Lu, Yingji Chen, Wei Feng

**Affiliations:** 1 Department of Cardiothoracic Surgery, Third Xiangya Hospital, Central South University, Changsha, 410013 Hunan China; 2 Center for Experimental Medicine, Third Xiangya Hospital, Central South University, Changsha, 410013 Hunan China

**Keywords:** Cancer models, Tumour biomarkers, Non-small-cell lung cancer, Prognostic markers, Neutrophils

## Abstract

Lung adenocarcinoma (LUAD) remains an incurable disease with a poor prognosis. This study aimed to explore neutrophil‑related genes (NRGs) and develop a prognostic signature for predicting the prognosis of LUAD. NRGs were obtained by intersecting modular genes identified by weighted gene co-expression network analysis (WGCNA) using bulk RNA-seq data and the marker genes of neutrophils identified from single-cell RNA-sequencing(scRNA-seq) data. Univariate Cox regression, least absolute shrinkage and selection operator (LASSO), and multivariate Cox analyses were run to construct a prognostic signature, follow by delineation of risk groups, and external validation. Analyses of ESTIMAT, immune function, Tumor Immune Dysfunction and Exclusion (TIDE) scores, Immune cell Proportion Score (IPS), and immune checkpoint genes between high- and low-risk groups were performed, and then analyses of drug sensitivity to screen for sensitive anticancer drugs in high-risk groups. A total of 45 candidate NRGs were identified, of which PLTP, EREG, CD68, CD69, PLAUR, and CYP27A1 were considered to be significantly associated with prognosis in LUAD and were used to construct a prognostic signature. Correlation analysis showed significant differences in the immune landscape between high- and low-risk groups. In addition, our prognostic signature was important for predicting drug sensitivity in the high-risk group. Our study screened for NRGs in LUAD and constructed a novel and effective signature, revealing the immune landscape and providing more appropriate guidance protocols in LUAD treatment.

## Introduction

Lung carcinoma is the second most commonly diagnosed cancer and the leading cause of cancer death in recent years^1^. It is divided into two main subtypes: small-cell lung carcinoma (SCLC) and non-small-cell lung carcinoma (NSCLC)^2^. Among NSCLC, lung adenocarcinoma (LUAD) is the most common type in all newly diagnosed cases^3^. LUAD patients with early stage can receive standard surgical treatment, but the vast majority of patients are usually diagnosed at an advanced stage, with a low 5-year survival rate. The tumor microenvironment (TME) is a complex micro-ecosystem composed of tumor cells, immune cells, inflammatory cells, microvasculature, and extracellular matrix (ECM)^4^. The heterogeneity of immune cell infiltration is a key factor influencing immune response and prognosis in LUAD and other tumors^5,6^. Therefore, the prognostic signature based on specific immune cell biomarkers can predict the immune response and survival more accurately.

Neutrophils, the most abundant circulating cells in human blood, play a crucial role in fighting infections and maintaining dynamic tissue homeostasis^7,8^. With the advancement of technology in recent years, such as in vivo imaging, high-dimensional transcriptomic and epigenomic approaches, and single-cell RNA-sequencing (scRNA-seq), the awareness of neutrophils is no longer limited to the inflammatory response and adaptive immunity, but it has also been found to play crucial roles in the growth and development of tumors. Neutrophils can release reactive oxygen species (ROS) and induce oxidative DNA damage in tissues, thereby promoting tumorigenesis^9,10^. Neutrophils also directly support the proliferation of tumor cells through various paracrine signaling pathways^11,12^. In addition, neutrophils can protect cancer cells from cytotoxic immune cells by inhibiting the killing ability of other immune cells and releasing neutrophil extracellular traps (NETs)^13,14^. Neutrophils are also closely associated with lung cancer. In RAS-driven lung cancer, neutrophils can release elastase to degrade insulin receptor substrate 1 and promote cancer cell proliferation^12^. In SOX2 overexpressing non-small cell lung cancer models, CXCL58-dependent TANs (Tumer-associated neutrophils) can promote tumor growth and squamification^15^. Neutrophils can also induce zinc finger protein expression, which in turn promotes the epithelial-mesenchymal transition of cancer cells in a mouse model of lung cancer by mediating T-cell rejection and hypoxia^16^. However, the relationship between neutrophils and the prognosis of lung adenocarcinoma is unclear. There is mounting evidence suggesting the role of neutrophils in LUAD progression, supporting the need for further studies to clarify this relationship.

In this study, we combined scRNA-seq and conventional bulk RNA sequencing analysis to construct a novel and accurate 6-gene LUAD prognostic signature. Our signature effectively predicts the prognosis of LUAD patients and reveals a potential link between risk characteristics, tumor microenvironment, immunotherapy, and drug sensitivity.

## Results

### Screening for genes associated with neutrophil content by weighted gene co-expression network analysis (WGCNA)

**Figure 1:**
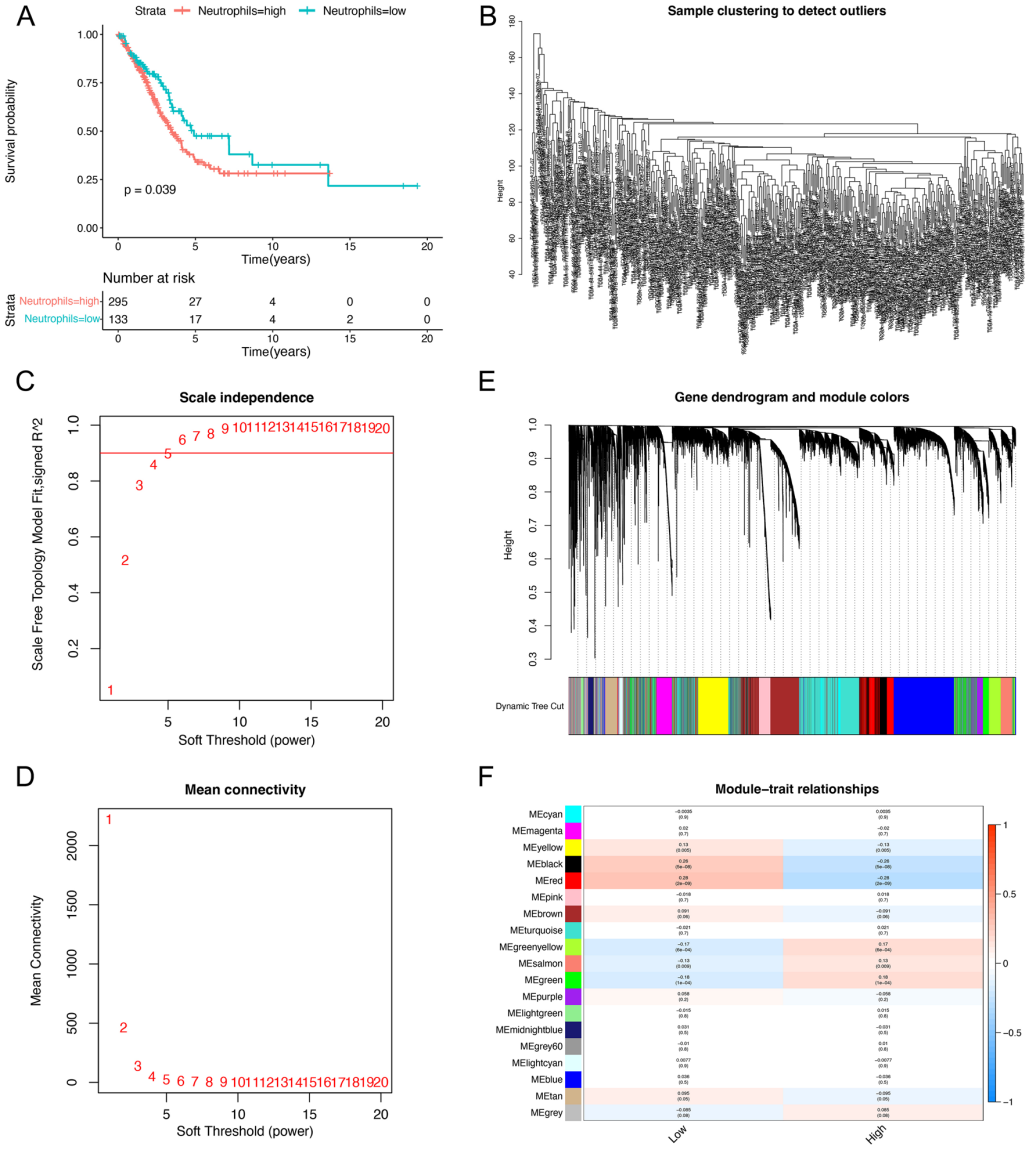
Neutrophil-related survival analysis and screening of neutrophil content-related genes by WGCNA. (**A**) Kaplan–Meier survival curves showed that the prognosis was significantly worse in the group with high content of neutrophils. (**B**) TCGA-LUAD samples were clustered and outlier samples were not found. (**C,D**) Based on the near scale-free network distribution criterion, 5 was selected as the soft threshold power. (**E,F**) Correlation analysis of modules with traits constructed 18 non-gray modules, and the green module is considered to be the most relevant module for neutrophils.

Before constructing the WGCNA co-expression network, we wished to further elucidate the relationship between neutrophils and the prognosis of LUAD patients. The mRNA transcriptomic data and clinical information of 555 LUAD patients were downloaded from The Cancer Genome Atlas Program (TCGA, https://www.cancer.gov/) database, and 473 eligible samples were obtained after excluding duplicate samples, normal samples, and samples with incomplete information on survival status and time. We used the “CIBERSORT” package to calculate the relative neutrophil content of each sample in TCGA-LUAD and then divided the TCGA-LUAD patients into high- and low-neutrophil-content groups based on the best cut-off value (cut. off = 0.001917716). Kaplan–Meier survival analysis showed that LUAD patients in the high neutrophil content group had a lower survival rate (Fig. 1A). It suggested that neutrophils probably play an important role in the prognosis of LUAD patients. Based on this result, we used the “WGCNA” package to construct the co-expression network to screen for genes significantly associated with neutrophil content in LUAD. First, no outliers were detected in the TCGA-LUAD samples (Fig. 1B), and the soft threshold power *β* was 5 when the fit index of the scale-free topology reached 0.90 (Figs. 1C, D). Based on the average linkage hierarchical clustering and soft threshold power, we identified a total of 19 gene modules (Fig. 1E). Correlation analysis between gene modules and neutrophil content showed that the green module had the most significant correlation with high neutrophil content (correlation = 0.18, *p*-value < 0.001) (Fig. 1F). Therefore, we selected 1277 genes from the green module for subsequent analysis (Supplementary Table S3).

### Identification of neutrophil marker genes

**Figure 2:**
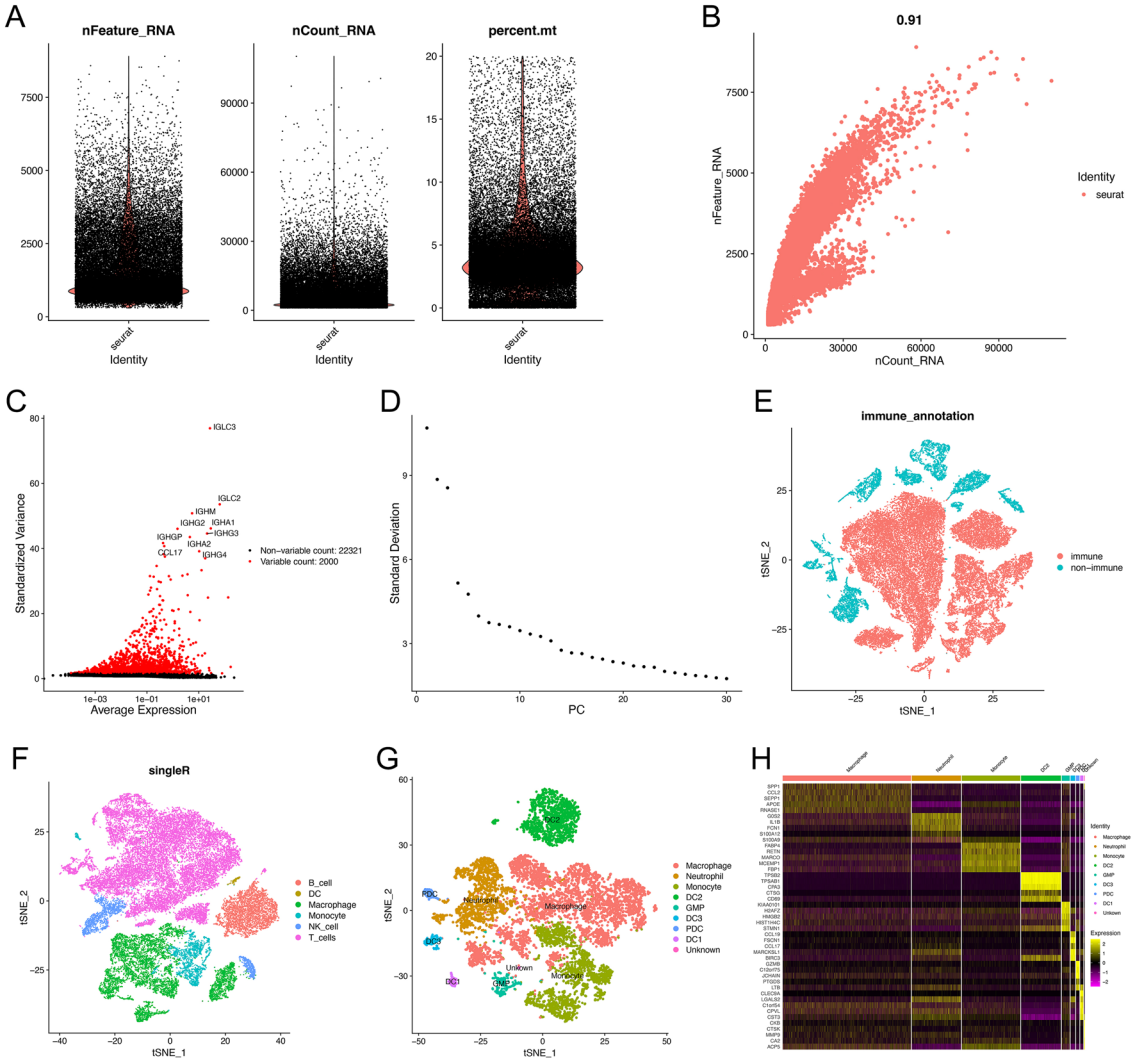
Single-cell analysis and acquisition of neutrophil marker genes. (**A**) Quality control of scRNA-seq data of GSE131907. (**B**) The number of genes detected was positively associated with the depth of sequencing. (**C**) Scatter plots showed the top 2000 highly variable genes. (**D**) Principal component analysis was employed for dimensionality reduction, and the suitable PCs were selected by elbow point. (**E**) First, 42,431 cells were annotated as “immune cells” and “non-immune cells” by the t-SNE algorithm. (**F**) Second, further detailed annotation of immune cells. (**G**) Finally, 13 clusters were obtained after the thirst-level classification of myeloid immune cells, and 9 cell types were identified by marker gene annotation. (**H**) Heatmap demonstrated the marker genes with differential expression in 9 types of cells.

After data processing and quality control, we obtained gene expression profiles of 42,431 cells of 11 primary LUAD samples from the Gene Expression Omnibus (GEO, https://www.ncbi.nlm.nih.gov/geo/) database. The number of genes(nFeature), the sequence count per(nCount), and the percentage of mitochondrial genes(percent. mt) were displayed in Fig. 2A. Correlation analysis showed that nCount was positively correlated with nFeature (Fig. 2B). We performed principal component analysis (PCA) to reduce dimensionality based on 2000 highly variable genes and visualized the top 10 genes with the most significant change (Fig. 2C). We selected the top 20 principal components (PCs) for t-SNE analysis using the elbow plot (Fig. 2D) and identified 28 different clusters. First, we classified all cells into immune (N = 33,524) and non-immune (N = 8907) cells (Fig. 2E) based on the level of gene PTPRC (CD45) expression. We then performed the second downscaling of all immune cells to obtain 22 clusters, which were cell annotated using the “SingleR” package. After this step, we identified six cell types, including B cells, T cells, natural killer (NK) cells, monocytes, macrophages, and dendritic cells (DCs) (Fig. 2F). We performed further subgroup clustering of myeloid immune cell populations (monocytes, macrophages, DCs). The myeloid immune cells were classified into 13 clusters and next, we defined cellular annotations for each cluster by cross-referencing differentially expressed genes in each cluster with typical marker genes obtained from the CellMarker database and the PanglaoDB database. Finally, we identified nine cell types (Fig. 2G), including macrophages, neutrophils, monocytes, granulocyte–macrophage progenitors (GMP), plasmacytoid dendritic cells (PDCs), DC1, DC2, DC3, and unknown cells, and cluster 1 was defined as neutrophils. Information on the annotation of myeloid immune cell subpopulations is shown in the Supplementary Table S4. Overall, after the above steps, we ultimately identified 88 neutrophil marker genes for LUAD (Supplementary Table S5), and shown in the heatmap (Fig. 2H).

### Functional enrichment of neutrophil‑related genes (NRGs)

**Figure 3:**
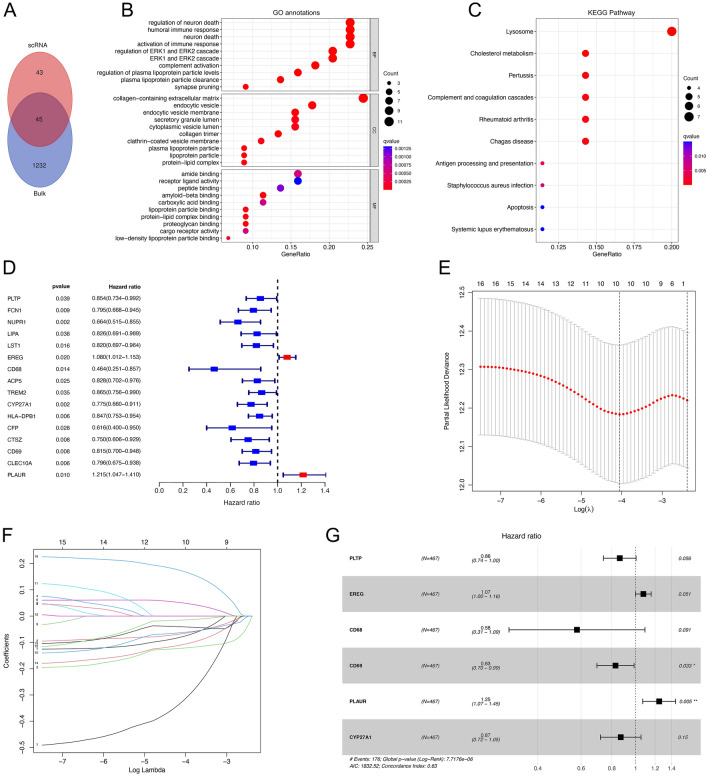
Functional enrichment analysis of neutrophil-related genes and construction of a neutrophil-related prognostic signature. (**A**) Acquisition of candidate NRGs. (**B,C**) Enrichment analysis using the GO database and KEGG database. (**D**) Forest plots showed the prognostic value detection of neutrophil-related genes. (**E,F**) Lasso regression analysis to identify signature genes and 16 neutrophil-related genes were selected to construct the LASSO model. (**G**) 6 genes which were selected by stepwise multiple COX regression analysis for the neutrophil-related prognostic model.

We obtained 45 candidate NRGs (Fig. 3A, Supplementary Table S6) after taking intersections of 1277 neutrophil module genes and 88 neutrophil marker genes. According to the Gene Ontology (GO, http://geneontology.org/) database annotation, NRGs were significantly enriched in 670 items, of which the top 10 were shown in the bubble map (Fig. 3B). The biological process (BP) categories mainly included “regulation of neuron death”, “humoral immune response”, “neuron death” and “activation of immune response”, etc. The cellular component (CC) categories included mainly “collagen-containing extracellular matrix” and “endocytic vesicle”. The Kyoto Encyclopedia of Genes and Genomes (KEGG, https://www.genome.jp/kegg/) functional enrichment analysis showed that NRGs were significantly enriched in 16 pathways. The top 10 were shown in the bubble diagram (Fig. 3C) and mainly involved in “Lysosome”, “Cholesterol metabolism”, “Complement and coagulation cascades” and “coagulation cascades” pathways.

### Construction of the risk model based on NRGs in LUAD

First, we used the TCGA cohort as the training set and the “survival” package for univariate COX regression analysis to obtain prognosis-related genes. As shown in the forest plot (Fig. 3D), 16 NRGs were obtained that were significantly associated with overall survival (OS) (*p*-value < 0.05). The least absolute shrinkage and selection operator (LASSO) regression analysis was then used to screen for key prognostic genes, and ten NRGs were identified (Figs. 3E, F) through this step by performing 1000 variable screens and resampling on these 16 genes and selecting those with more than 900 replicates. Next, we identified six stable prognostic NRGs and their regression coefficients (Fig. 3G) by stepwise multiple COX regression analysis. We eventually constructed a prognostic risk model containing six NRGs and calculated risk scores according to the following equation, and the regression coefficients of six genes included in the prognostic model are shown (Supplementary Table S7):

$$risk \, score=-0.1489*PLTP \, expression \, + \, 0.0722*EREG \, expression \, - \, 0.5497*CD68 \, expression-0.1891*CD69 \, expression+0.2204*PLAUR \, expression-0.1398*CYP27A1 \, expression$$

**Figure 4:**
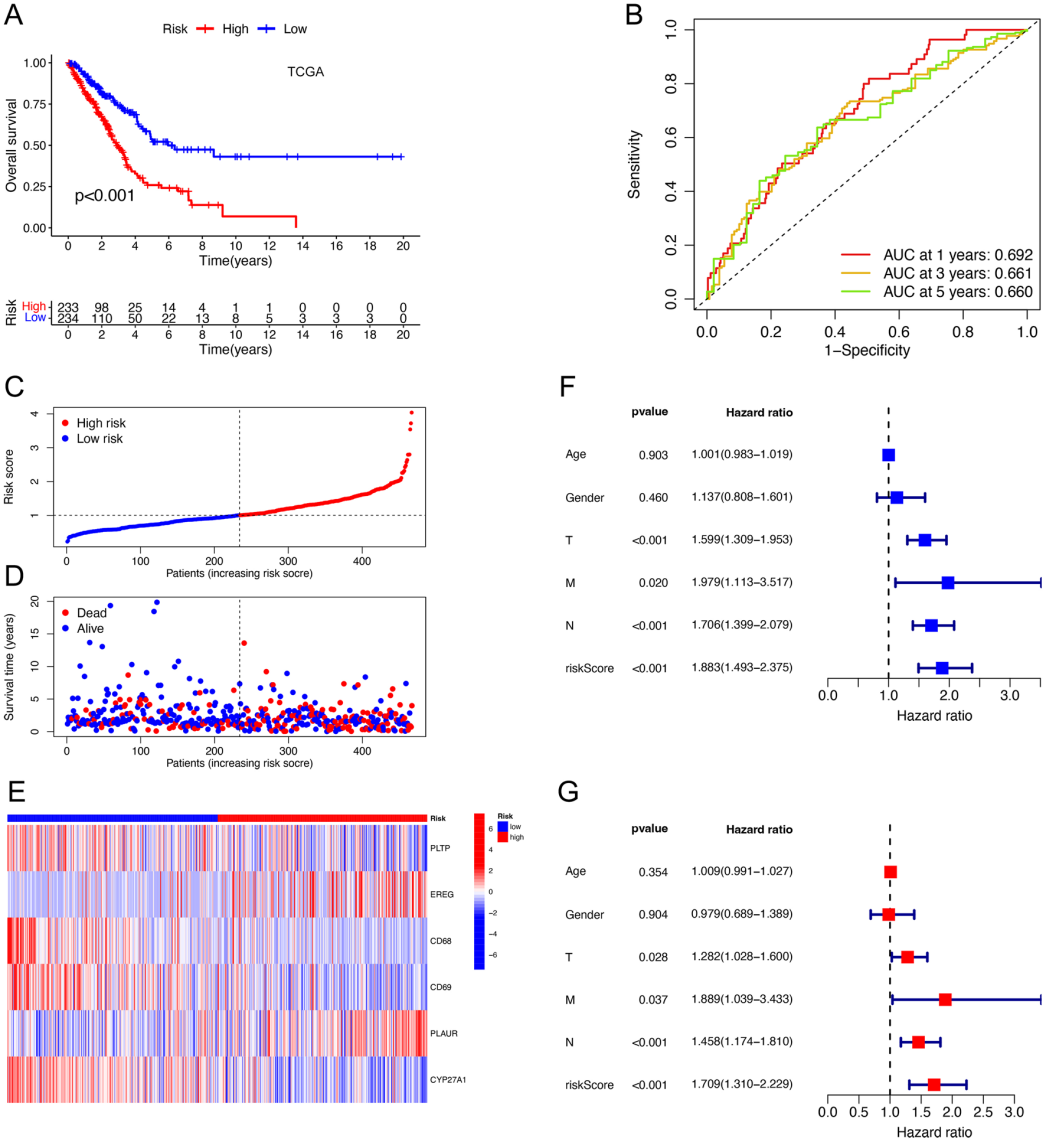
Identification of the prognostic six-gene risk signature in the TCGA cohort. (**A**) Kaplan–Meier curves of overall survival probability of risk groups in TCGA cohort. (**B**) ROC curves for 1, 3, and 5 years and their AUCs in the TCGA cohort. (**C,D**) Risk scores and survival status distribution of LUAD patients in high- and low-risk groups in TCGA cohort. (**E**) Heatmap showed the expression difference for 6 neutrophil-related genes among the risk groups in TCGA. (**F,G**) Forest plots of univariate and multivariate Cox regression analyses revealed that risk score could be an independent prognostic factor.

**Figure 5:**
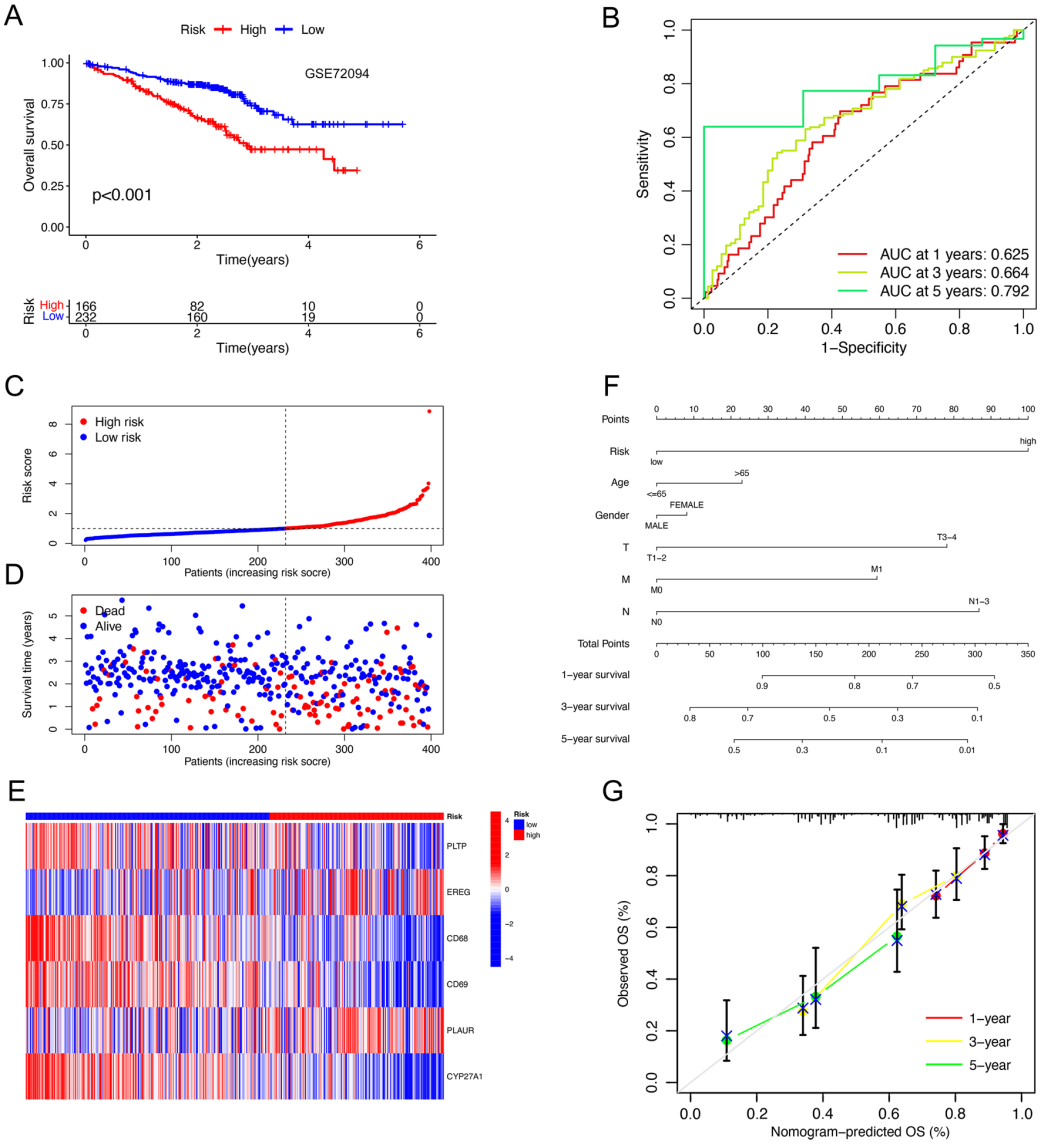
Validation of the prognostic six-gene risk signature in GSE72094 and construction of the nomogram. (**A**) Kaplan–Meier curves of overall survival probability of risk groups in GSE72094. (**B**) ROC curves for 1, 3, and 5 years and their AUCs in GSE72094. (**C,D**) Risk scores and survival status distribution of LUAD patients in high- and low-risk groups in GSE72094. (**E**) Heatmap showed the expression difference for 6 neutrophil-related genes among the risk groups in GSE72094. (**F**) Nomogram of TCGA cohorts based on the risk scores and other clinical characteristics. (**G**) Calibration graphs investigated that the actual survival rates of LUAD patients were close to the nomogram-predicted survival rates.

Based on the median risk score, we divided the TCGA-LUAD samples into high-risk and low-risk groups. Kaplan–Meier survival curves (Fig. 4A) showed that patients in the low-risk group had a better overall prognosis than those in the high-risk group (*p-*value < 0.001). The "timeROC" package was used to construct time-dependent receiver operating characteristic (ROC) curves with area under curve (AUC) of 0.692, 0.661, and 0.660 for 1, 3, and 5 years (Fig. 4B) respectively in the TCGA cohort. The risk curves and survival status plots (Fig. 4C, D) showed that the higher the risk score, the lower the survival rate and the higher the number of deaths. The risk heat map (Fig. 4E) suggested that the expression of EREG and PLAUR was higher in the high-risk group than in the low-risk group, while the expression of PLTP, CD68, CD69, and CYP27A1 was relatively lower. The univariate Cox regression and multivariate COX regression analyses (Figs. 4F, G) suggested that tumor stage and risk score were independent prognostic factors for poor survival. Excluding samples with incomplete information on survival time and survival status, a total of 398 LUAD samples from GSE72094 were used for external validation of the risk model. Consistently, we observed similar results (Fig. 5A–E) in the GSE72094 cohort, all suggesting that this prognostic risk model had excellent predictive power for the survival prognosis of LUAD patients. In addition, we combined risk scores and different clinicopathological characteristics to construct the Nomogram (Fig. 5F). Based on the calibration curves for OS at 1, 3, and 5 years (Fig. 5G), the predicted and actual survival rates of this nomogram were very close, suggesting that the nomogram has good predictive value in predicting OS of LUAD patients.

### Correlation of high- and low-risk groups with clinicopathological characteristics

**Figure 6:**
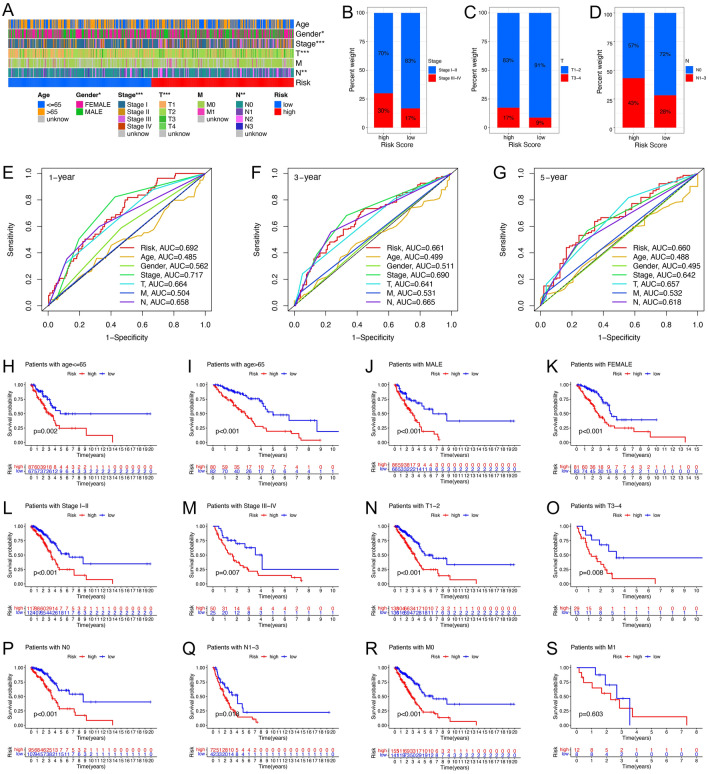
Correlations analysis between the risk signature and clinicopathological characteristics. (**A**) Heatmap demonstrated the differences in clinicopathological features between high- and low-risk groups in the TCGA cohort. (**B–D**) The bar plot showed the correlation between risk score and Stages (**B**), T (**C**), and N (**D**) of LUAD. (**E–G**) ROC curves for 1-year (**E**), 3-year (**F**), and 5-year (**G**) survival prediction, and clinicopathological characteristics. (**H–S**) Kaplan–Meier plots depicted subgroup survival analysis stratified by age (**H,I**), sex (**J,K**), tumor stage (**L,M**), T (**N,O**), M (**P,Q**), and N (**R,S**). *p-value < 0.05, **p-value < 0.01, ***p-value < 0.001.

The clinicopathological characteristics of patients in the high- and low-risk groups in the TCGA cohort were shown in Fig. 6A. The risk scores were significantly correlated with clinical characteristics such as tumor stage, T-stage, and N-stage (Figs. 6B–D). The time-dependent ROC curves (Figs. 6E–G) constructed based on the clinicopathological characteristics showed that the AUC of the risk score predicting 1-year and 3-year survival was 0.692 and 0.661, respectively, which were close to the AUC of tumor stage, and the AUC of the risk score predicting 5-year survival was 0.660, which was slightly higher than the AUC of tumor stage predicting 5-year survival. To further investigate the relationship between prognosis and risk score in different clinicopathological characteristics, we divided patients into two different subgroups according to age (Fig. 6H, I), gender (Fig. 6J, K), tumor stage (Fig. 6L, M), T stage (Fig. 6N, O) M stage (Fig. 6P, Q) and N stage (Fig. 6R, S). We found that higher risk scores were significantly associated with Stage III–IV, T3–4, and N1–3 stages, and OS was longer for patients in the low-risk group than for those in the high-risk group in most clinical subgroups (except for patients in the M1 subgroup). These results suggested that the prognostic model remains a strong predictor for patients with different clinicopathological characteristics.

### Differences in the tumor microenvironment and gene set enrichment between high- and low-risk groups

**Figure 7:**
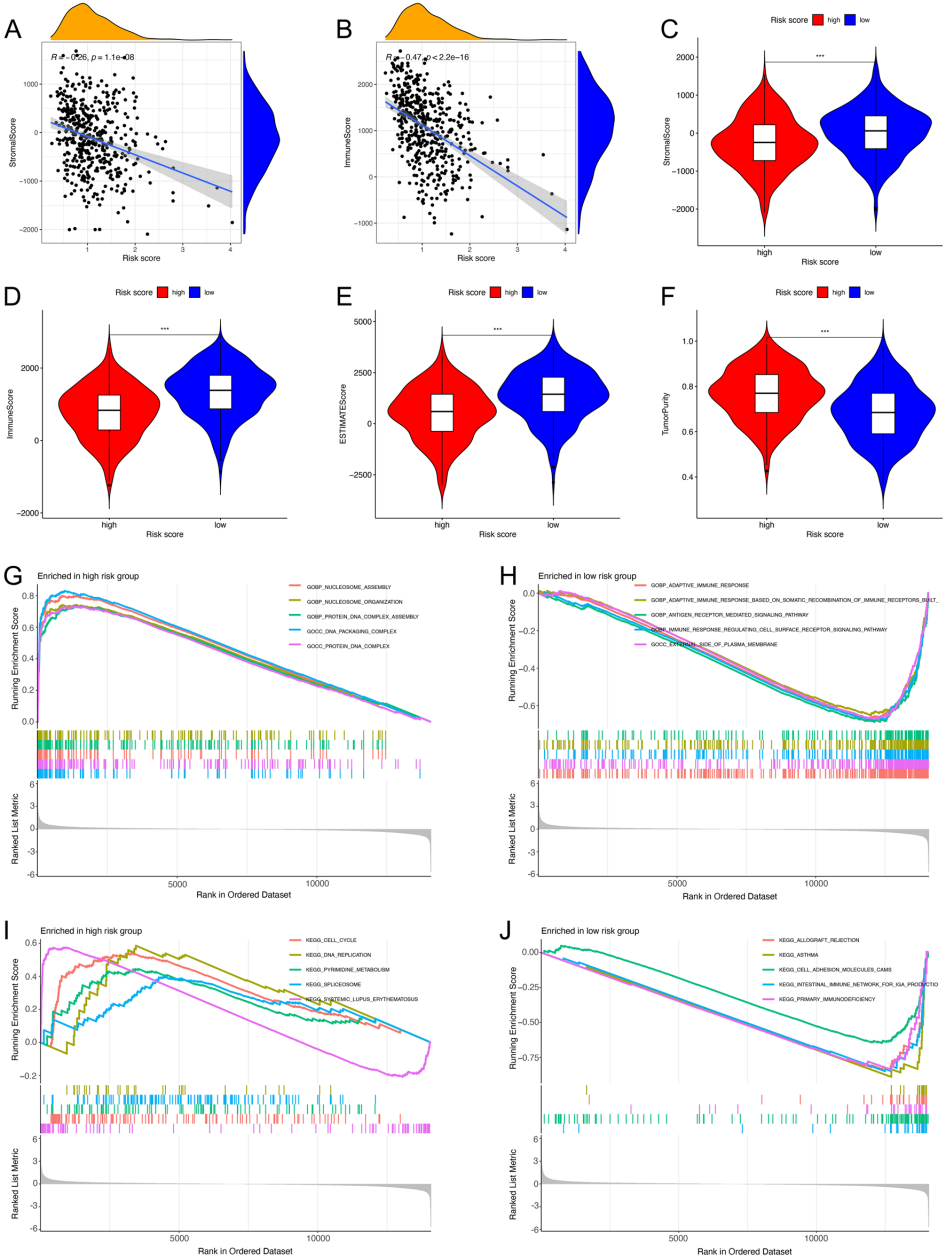
Evaluation of tumor microenvironment and gene set enrichment analysis in high- and low-risk groups. (**A**) Spearman analysis between stromal score and risk score. (**B**) Spearman analysis between immune cell score and risk score. (**C–F**) Violin plots showed the differences in the stromal score (**C**), immune scores (**D**), estimate scores (**E**), and tumor purity (**F**) in high- and low-risk groups. *p-value < 0.05, **p-value < 0.01, ***p-value < 0.001. (**G,H**) Top enriched GO functions in high- (**G**) and low-risk groups (**H**). (**I,J**) Top enriched KEGG pathways in high- (**I**) and low-risk groups (**J**).

We further investigated the correlation between risk scores and ESTIMATE-related scores. We found a negative correlation between Immune, Stromal, and ESTIMATE scores with risk scores (Fig. 7A–E), and a positive correlation between tumor purity and risk scores (Fig. 7F). To identify pathways and functions that were significantly enriched between high- and low-risk groups, we performed gene set enrichment analysis (GSEA). In terms of GO function, the high-risk group focused on “nucleosome assembly”, “nucleosome organization”, “DNA packaging complex”, “protein DNA complex” and “protein DNA complex assembly” (Fig. 7G). The low-risk group focused on “adaptive immune response”, “antigen receptor-mediated signaling pathway” and “the external side of plasma membrane” (Fig. 7H). In terms of KEGG pathways, mRNAs in the high-risk group were significantly enriched in “cell cycle”, “DNA replication”, “pyrimidine metabolism” and “spliceosome” pathways (Fig. 7I). However, mRNAs in the low-risk group were significantly enriched in “allograft rejection”, “cell adhesion molecules cams” and “primary immunodeficiency” pathways (Fig. 7J).

### Immune landscapes and immunotherapy

**Figure 8:**
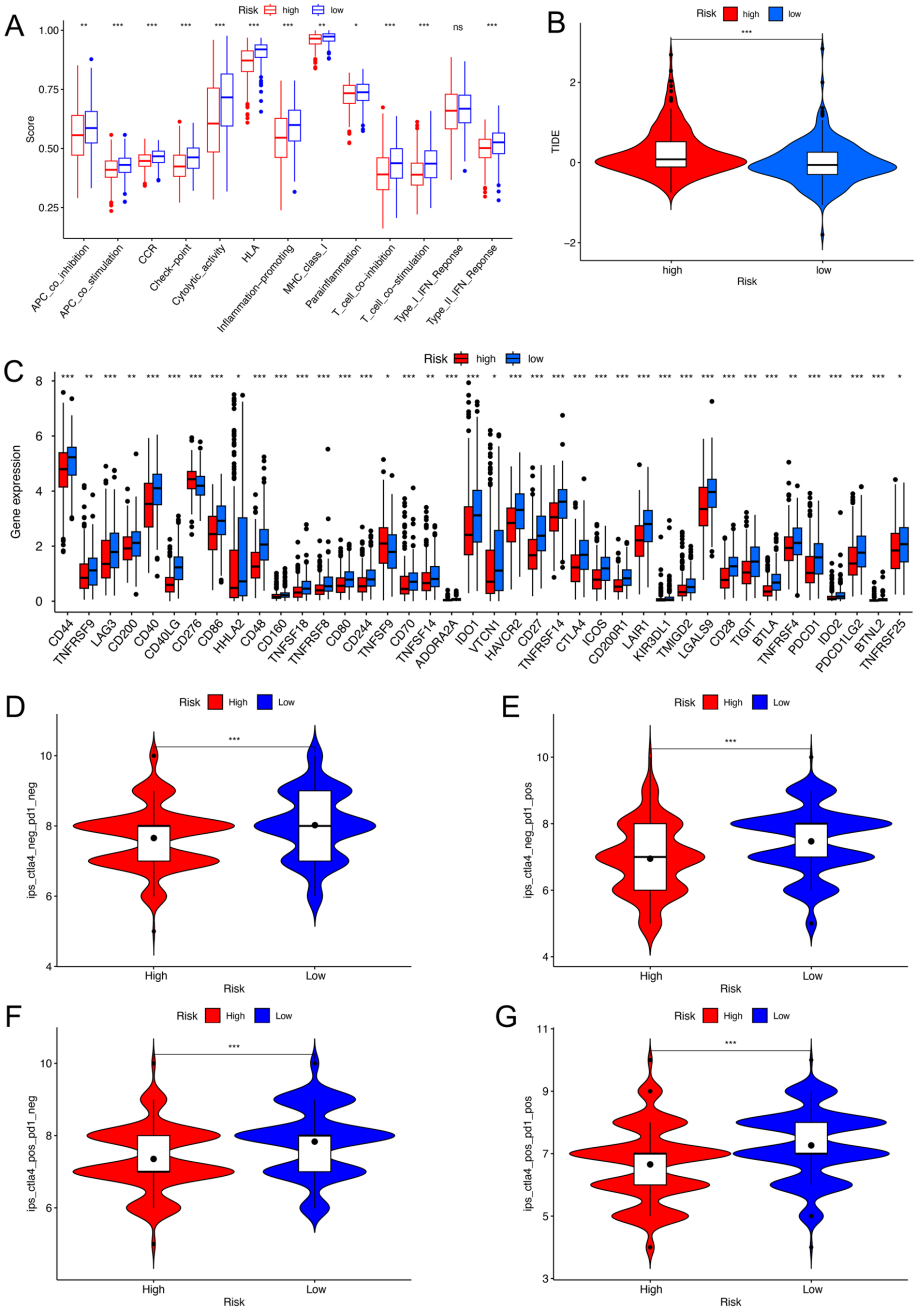
Risk signature-related immune landscapes. (**A**) The ssGSEA scores of 13 immune-related functions in high- and low-risk groups. (**B**) TIDE scores of high- and low-risk groups. (**C**) The differences of immune checkpoint gene expression in high- and low-risk groups. (**D–G**) Violin plots showed the differences in IPS among different risk groups in the four situations: negative immunoresponse to both PD-L1/PD-1 inhibitors and CTLA-4 inhibitors (**D**); positive immunoresponse to PD-L1/PD-1 inhibitors (**E**); positive immunoresponse to CTLA-4 inhibitors (**F**); positive immunoresponse to both PD-L1/PD-1 inhibitors and CTLA-4 inhibitors (**G**). *p-value < 0.05, **p-value < 0.01, ***p-value < 0.001.

We used single sample gene set enrichment analysis(ssGSEA) to evaluate the activity of immune-related pathways in different risk groups. It was clear that there were significant differences (Fig. 8A) in the vast majority of immune-related function scores between the high- and the low-risk groups (except for TYPEIIFNREPONSE), indicating a strong correlation between high-risk phenotype and immunosuppression. We also studied the relationship between risk scores and gene expression levels in immune checkpoints. Correlation analysis (Fig. 8C) showed that, except for CD276 and TNFSF9, most immune checkpoint genes were up-regulated in low-risk groups. In the aspect of immunotherapy, immune checkpoint blockade (ICB) therapy represented by Programmed Death 1 (PD-1) and Cytotoxic T Lymphocyte Antigen-4 (CTLA4) blockers is an effective means to treat tumors at present. We obtained IPS (Immune cell Proportion Score) for each TCGA-LUAD patient from The Cancer Immune Atlas (TCIA, https://www.tcia.at/home) database to explore the role of risk scores in predicting response to immunotherapy. We found that the overall average value of IPS in the low-risk group was significantly higher than that in the high-risk group, whether in tumor samples that were predicted to be negative for both immunotherapy regimens (Fig. 8D) or in samples that were positive for a single regimen (Fig. 8E, F) or both regimens (Fig. 8G). In addition, we found that the Tumor Immune Dysfunction and Exclusion (TIDE) score (Fig. 8B) of the low-risk group was lower than that of the high-risk group, indicating that patients in the low-risk group were more likely to benefit from immunotherapy.

### Selection of targeted and chemotherapeutic agents suitable for patients in the high-risk group

**Figure 9:**
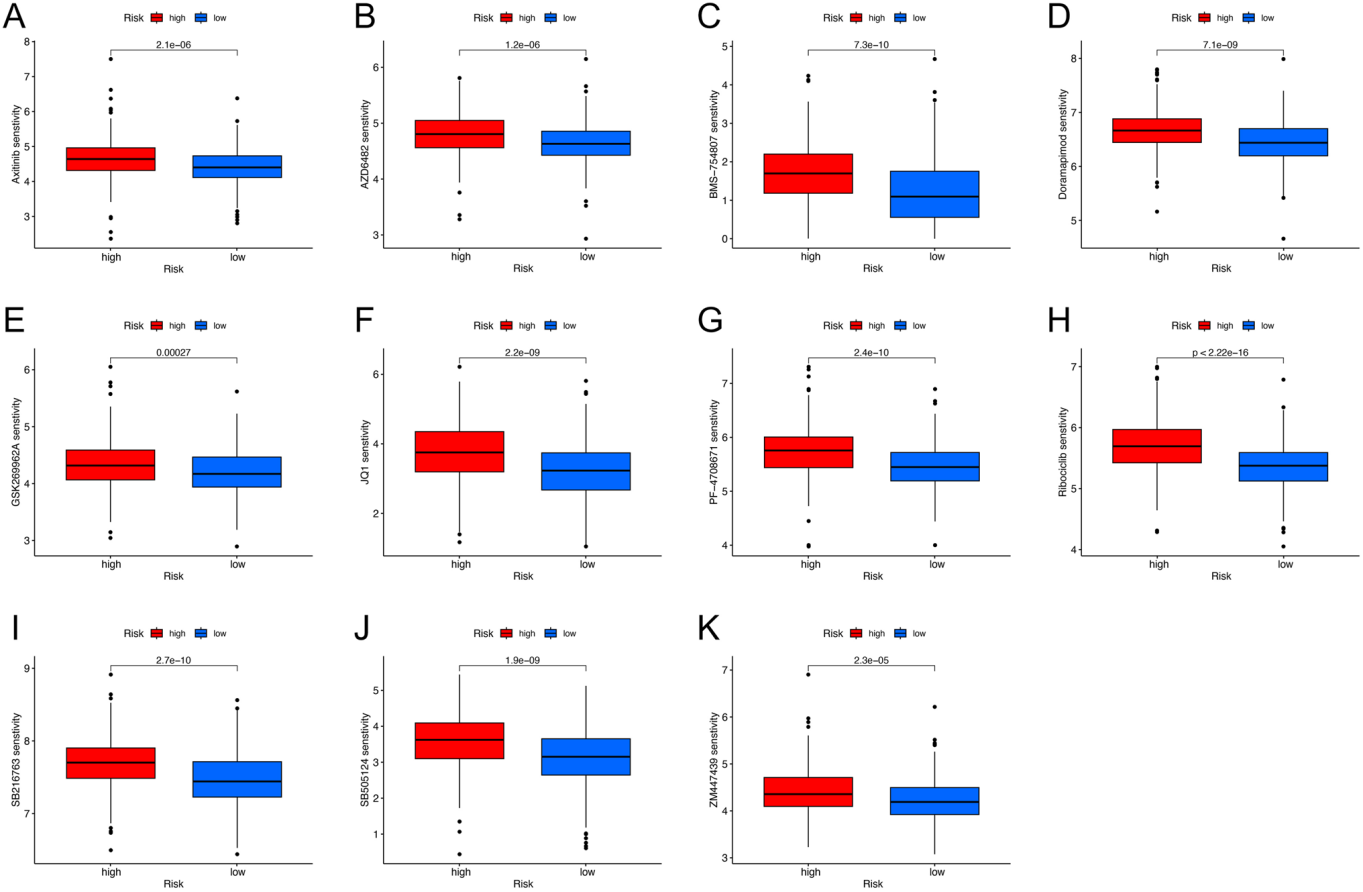
Predicted sensitivity scores of drugs that are candidate therapeutic agents for high-risk LUAD patients. (**A**) Axitinib, (**B**) AZD6482, (**C**) BMS-754807, (**D**) Doramapimod, (**E**) GSK269962A, (**F**) JQ1, (**H**) PF-4708671, (**I**) Ribociclib, (**J**) SB216763, (**K**) SB505124, (**L**) ZM447439.

To explore targeted and chemotherapeutic agents for patients in high-risk groups, we translated the LUAD-TCGA gene expression profile into a drug sensitivity matrix by the “oncopredict” package. The drug sensitivity results suggested that a total of 32 drugs differed significantly between high- and low-risk groups. Among these, the high-risk LUAD patients were more sensitive to 11 drugs (Fig. 9A–K), including Axitinib(VEGFR inhibitor), AZD6482(PI3Kβ inhibitor), BMS-754807(IGF-1R/IR inhibitor), Doramapimod(p38 MAPK inhibitor), GSK269962A(ROCK inhibitor), JQ1(BET BRD inhibitor), PF-4708671(S6K1 inhibitor), Ribociclib(CDK4/6 inhibitor), SB216763(GSK-3 inhibitor), SB505124(ALK4/5/7 inhibitor), ZM447439(Aurora inhibitor).

### Validation of signature genes in LUAD tissue

**Figure 10:**
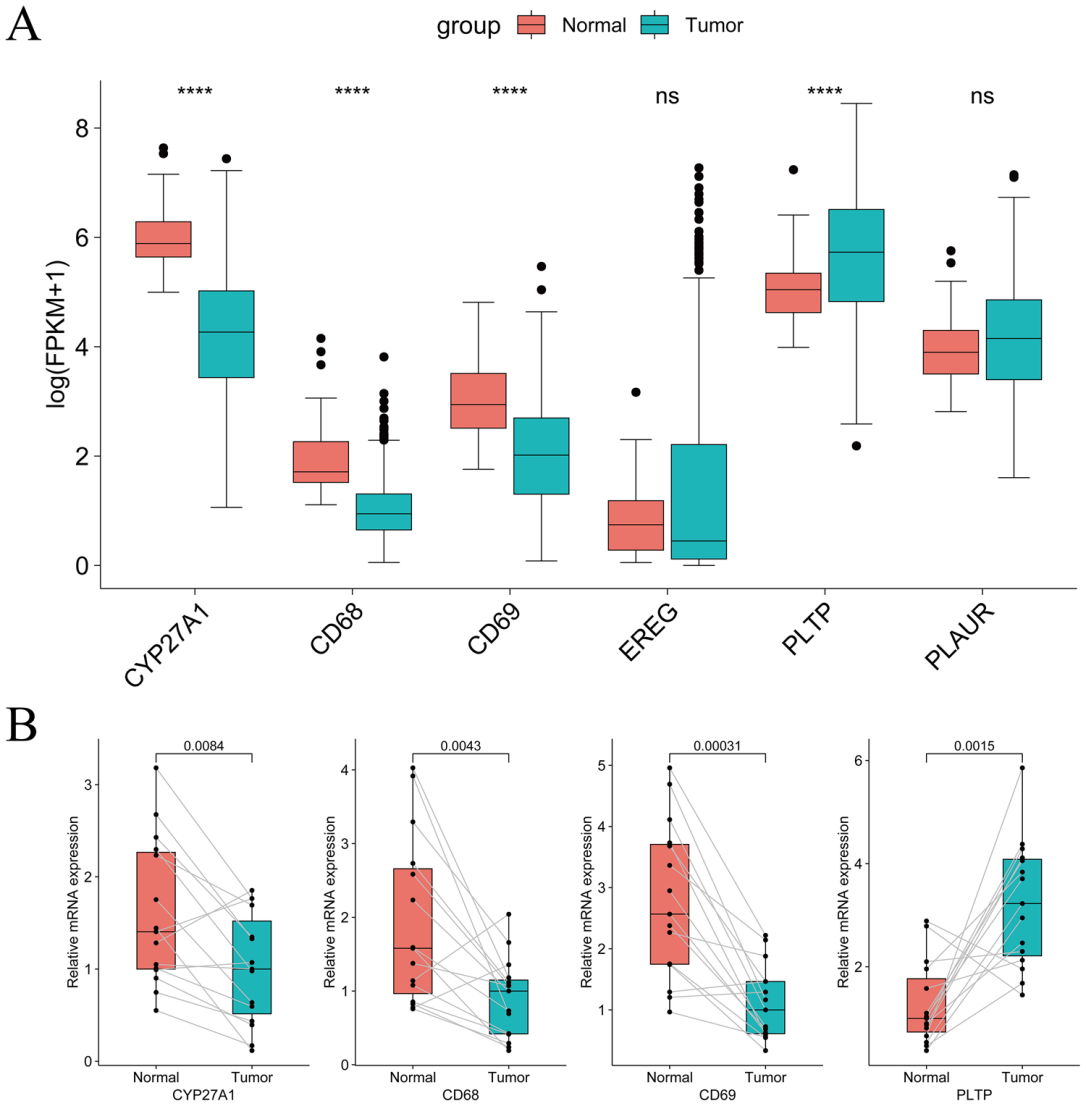
Measure the signature genes expression in the LUAD tissue. (**A**) The signature gene expression in TCGA-LUAD samples (normal = 54, tumor = 501). (**B**) The mRNA expression of CYP27A1, CD68, CD69, and PLTP in 15 primary tumor tissues and 15 adjacent normal tissues. Statistics were considered significant when the *p-value was less than 0.05, **p-value less than 0.01, ***p-value less than 0.001 and ****p-value less than 0.0001.

After obtaining the NRGs and constructing a NRGs-related prognostic signature, we further analyzed the expression of signature genes in the TCGA-LUAD samples and the samples we obtained from LUAD patients. Figure 10A showed that the expression of CD68, CD69 and CYP27A1 were significantly downregulated, and the expression of PLTP were significantly upregulated in tumor samples. Similarly, the qRT‐PCR results showed that the mRNA expression levels of these four genes have significant differences in tumor tissues and adjacent normal tissues (Fig. 10B).

## Discussion

LUAD is the main pathological type of NSCLC, which is considered to be related to a variety of immune cell infiltration in the lung tumor microenvironment^17^. The tumor microenvironment refers to the surrounding microenvironment of tumor cells, including blood vessels, immune cells, fibroblasts, bone marrow-derived inflammatory cells, various signal molecules, and ECM^18^. In the past decade, the central role of tumor microenvironment in the occurrence and development of primary lung cancer has been recognized^17,19,20^. In addition, extrathoracic malignant tumors, including breast cancer, colon cancer, and melanoma, reprogram the lung microenvironment to support the colonization and growth of spreading tumor cells, resulting in secondary lung tumors^21^.

Neutrophils are one of the myeloid immune cells and an important component of the tumor microenvironment. In recent years, neutrophils have received more and more attention. Several studies have found that neutrophils are not only involved in the initiation and regulation of inflammation and immune response, but also play crucial roles in cancer progression and metastasis, via direct effects on cancer cells^10,12,22,23^, remodeling of the ECM^24,25^, stimulation of angiogenesis^16,26^, activation of protumorigenic macrophages^27^, inhibition of antitumor immunity^16,28^, production of ROS^10,26,29^, or release of NETs^14,30–32^. Therefore, we hope to further explore the prognostic relationship between neutrophils and LUAD, and establish an innovative prognostic signature.

In this study, we found that TCGA-LUAD patients with high neutrophil content had a significantly worse prognosis than those with low neutrophil content patients, which proved the correlation between neutrophils and the prognosis of LUAD. We combined the modular genes screened by WGCNA crossed with neutrophil marker genes obtained from single-cell sequencing analysis to obtain 45 NRGs. Ten prognosis-related genes were screened by univariate regression analysis and LASSO regression analysis, and the parameters and genes for the final prognosis model (PLTP, EREG, CD68, CD69, PLAUR, CYP27A1) were determined by stepwise multiple regression analysis.

Among these genes, some genes have been proven to be related to the occurrence and development of malignant tumors, but others have not been deeply studied. Plasma phospholipid transfer protein (PLTP) is a protein-encoding gene previously identified as a direct target gene of p53 in HepG2 cells^33^. The proteins it encodes play complex roles in multiple processes from tumor proliferation to immune function. Gnanapradeepan et al. found that PLTP was an effective inhibitor of cancer cell colony formation and played an important role in controlling the sensitivity of cells to iron death^33^. Desrumaux et al. found that PLTP modulated adaptive immune function by regulating the polarization of CD4+ T cells towards the pro-inflammatory Th1 type^34^. In addition, PLTP has recently been reported to regulate the phagocytic activity of macrophages and microglia, increase the production of the pro-inflammatory cytokine interleukin 6 (IL-6), and regulate the activation and degranulation of neutrophils^35^. Epidermal regulatory protein (EREG), one of the ligands of EGFR, is lowly expressed in most normal tissues. Elevated ERER levels can lead to aberrant activation of the epidermal growth factor receptor (EGFR/ERBB1), and the activated EREG/EGFR pathway further regulates various cellular functions, including cancer cell proliferation, survival, metastasis, and angiogenesis^36,37^. It has been reported that the EREG protein is significantly overexpressed in LUAD and associated with invasive tumor phenotype^38^. In bladder cancer, the expression of EREG in advanced patients is increased, which is related to the shorter OS^39^. In addition, the level of EREG protein in colorectal cancer is closely associated with tumor invasion and distant metastasis^40^. CD68 is a 110 KDa transmembrane glycoprotein, which is widely expressed in myeloid cell lines, including monocytes, macrophages, neutrophils, basophils, DCs, and myeloid progenitor cells^41^. In a CD68-associated pan-cancer analysis, CD68 was found to be highly expressed in many cancer types and associated with immune infiltration in tumor mutation burden (TMB), microsatellite instability (MSI), and TME, which may become a new immune checkpoint in future tumor immunotherapy^42^. CD69, a transmembrane type II C lectin protein^40^, is an early activation marker for a variety of leukocytes, including lymphocytes, granulocytes, macrophages, and dendritic cells, and plays an important role in the regulation of inflammatory and immune responses^43,44^. The role of CD69 in tumor immune response is controversial. For example, in melanoma patients, the expression of CD69 on memory CD8T cells in tumor antigen-specific tissues can prevent the growth and spread of cancer cells by promoting immune homeostasis^45^. In lung cancer patients who responded to PD-1/PD-L1 blocking, the expression of CD69 was upregulated, indicating that CD69 expression levels can effectively predict cancer response to PD-1/PD-L1 blockade immunotherapy^46^. Koyama-Nasu et al. found that anti-CD69 antibody could effectively inhibit the occurrence of lung metastasis and reduce the depletion of CD8T cells in tumor-bearing mice inoculated with 4T1 breast cancer^47^. In addition, it has been reported that anti-CD69 antibodies can enhance the anti-tumor effects against the murine renal cell carcinoma (Renca) cell line by promoting T cell proliferation, IL-2 expression, and cytotoxicity^48^. Thus, CD69 may have a double-edged sword effect on tumor immunity. Urokinase-type plasminogen activator receptor (PLAUR) is involved in a variety of biological processes, including angiogenesis, monocyte migration, cancer metastasis, trophoblast implantation, and wound healing. Its ligand uPA catalyzes plasminogen to form plasminogen and produces a proteolytic cascade, which contributes to tissue remodeling and ECM decomposition, and creates favorable conditions for tumor invasion and metastasis^49^. One study reported that PLAUR was significantly overexpressed in renal clear cell carcinoma and the level of PLAUR and PLAUR methylation was significantly correlated with poor prognosis and participated in the progression of renal clear cell carcinoma^50^. Similarly, Zhang et al. demonstrated that PLAUR can promote the growth and metastasis of gastric cancer and promote the loss of nesting apoptosis tolerance in gastric cancer cells by constructing the MKN45 gastric cancer mouse model and gastric cancer cell suspension anoikis model for 24 h^51^. CYP27A1 encodes a mitochondrial enzyme that is a member of the cytochrome P450 superfamily and is involved in the synthesis of bile acids, the oxidation of cytochrome P450, and the hydroxylation of cholesterol and vitamin D3. Liang et al. found that overexpression of CYP27A1 in bladder cancer cells can increase intracellular 27-HC production and reduce intracellular cholesterol levels. However, restoration of CYP27A1 expression inhibited the progression of T24 and UM-UC-3 bladder cancer cells, suggesting that CYP27A1 could inhibit bladder cancer cell proliferation by regulating cholesterol homeostasis^49^.Similarly, in patients with renal clear cell carcinoma, CYP27A1 could inhibit tumor proliferation and metastasis by activating the LXRs/ABCA1 axis^52^, which provides a new idea for anti-tumor therapy in the future.

The prognostic risk model was constructed according to the selected 6 NRGs, and the risk score of TCGA-LUAD patients was calculated. The patients were then divided into high- and low-risk groups based on median values. We found that patients in the high-risk group had a worse prognosis. The results of univariate regression analysis, multifactor regression analyses, and ROC curves suggest that our risk signature can predict the prognosis of LUAD independently of other indicators. We used GSE70294 as the test set to verify the prognostic signature externally, and the results were consistent with those of the TCGA-LUAD queue. Similarly, the prognostic signature remained applicable across different subgroups of clinicopathological features. The above results prove the universal applicability and validity of the signature. In addition, we constructed nomograms based on the signature and the clinicopathological indexes of the patients, The ROC curves of 1 year, 3 years, and 5 years illustrate its effectiveness in predicting OS in LUAD patients.

With the continuous understanding of TME, the view on the occurrence and development of cancer has gradually changed from tumor cells as the center to a complex tumor ecosystem that supports tumor growth, metastasis, and spread. Exploring differences in the tumor microenvironment can help guide and improve the role of various cancer therapies, particularly immunotherapies that act by enhancing the host's anti-tumor immune response^53^. In our study, the high-risk group had lower immune and interstitial scores and higher tumor purity than the low-risk group. It has been suggested that "hot" or inflammatory tumors show higher immunogenicity and tend to have a better immunotherapeutic response, according to the level of immune cell infiltration in the tumor microenvironment^54^. Therefore, the poor prognosis of patients in high-risk groups may be related to the immunosuppression of the LUAD tumor microenvironment. GSEA analysis shows that the mRNAs associated with the high-risk group were enriched in common tumor-related functions and pathways, such as nucleosome assembly, cell cycle, DNA replication, and so on. The mRNAs associated with the low-risk group were enriched in immune-related aspects, such as adaptive immune response and antigen receptor mediation. The results suggest that samples from the high-risk group may be more active in biological processes related to tumor development, which may contribute to the poorer prognosis of patients in the high-risk group.

In addition to effectively predicting the prognosis of LUAD patients, our study also found a significant correlation between risk groups and immune landscape and immunotherapy response. The results of ssGSEA showed that the scores of most immune function activities in the low-risk group were higher than those in the high-risk group. Immune checkpoint inhibitors (ICIs), including PD-1, PD-L1, and CTLA-4 inhibitors, can reactivate the anti-tumor response of the innate immune system by blocking the inhibitory immune checkpoint receptors present in TME or on tumor cells^55,56^. To further investigate the effect of immunotherapy in different risk groups, we compared the gene expression levels of immune checkpoints between the two groups. The majority of immune checkpoint gene expression levels are higher in the low-risk group than in the high-risk group, which means that patients in the low-risk group may have a stronger immune response and be more sensitive to immune checkpoint inhibitors. The IPSs of patients in both risk groups indicated that the patients in the low-risk group had a better response to PD-1 and CTLA-4 blocking therapy, The same results were found for the TIDE calculation system based on tumor immune escape mechanisms. The higher TIDE scores in the high-risk group indicate that these patients are more likely to experience immune escape and have poorer efficacy with ICB therapy. The above results confirm our conclusions based on TME correlation analysis. Therefore, the differences in the immune landscape revealed by the risk groupings based on our model suggest that the differences in the prognosis of LUAD patients may stem from heterogeneity in TME, which provides a new idea for our future research. In addition, we screened 11 targeted or chemotherapeutic agents that are sensitive to the high-risk group through drug sensitivity analysis. This evidence could also help guide chemotherapy and targeted therapy for high-risk LUAD patients. After constructing a prognostic signature, the expression of these genes in LUAD tissues remained unknown, We, therefore, measured their expression in the TCGA-LUAD samples and the tissues we collected. We found that the mRNA expression CD68, CD69 and CYP27A1 levels were significantly downregulated, and PLTP levels was significantly up regulated in tumor samples. These results suggested the signature genes may be potential therapeutic targets in LUAD.

However, our study still has some limitations. Firstly, the prognostic predictions and subsequent analyses in this study were based on data from the TCGA and GEO databases, and all samples were obtained retrospectively, which may lead to bias. Secondly, the stability of the signature performance needs to be confirmed in more prospective studies. Finally, the sensitivity of LUAD patients to chemotherapy and targeted agents also needs to be further validated in clinical studies with large samples.

In summary, we constructed a prognostic model composed of 6 NRGs through the comprehensive analysis of bulk RNA sequencing and single-cell sequencing, which can accurately predict the prognosis of LUAD patients. To some extent, this signature reveals the TME and immune landscape of LUAD, which can help guide more effective comprehensive treatment of LUAD patients.

## Methods

### Data source and acquisition

The single-cell mRNA expression file, GSE131907 was downloaded from the GEO database to screen neutrophil marker genes for LUAD. The mRNA transcriptome data of 555 LUAD patients (including 501 tumor samples and 54 normal paracancerous tissue samples) were downloaded from TCGA database. The gene expression data of TCGA-LUAD were downloaded and analyzed in the format of fragments per kilobase per million (FPKM). To verify the predictive effect of the constructed model on the prognosis of LUAD patients, the dataset containing 442 LUAD samples, GSE72094, was downloaded from the GEO database for external validation of the prognostic models. A summary of the clinicopathological characteristics of patients from TCGA and GEO database is shown in Supplementary Table S1. This study used publicly available datasets that had received ethical approval from the original study Each participant received informed consent.

### Neutrophil infiltration and related survival analysis

To explore the relationship between neutrophil content and survival of LUAD patients, we calculated the relative neutrophil content of each TCGA-LUAD sample by the “CIBERSORT” package^57^. The “survminer” package (https://rdocumentation.org/packages/survminer/), and the “surv-cutpoint” function were used to calculate the optimal cut-off value (cut. off) to distinguish between the high and low neutrophil content groups in the TCGA-LUAD samples. Survival analysis was carried out using the "survival" package (https://cran.r-project.org/web/packages/survival/index.html) and the Kaplan-Meier method was used to analyze and compare survival differences between the high and low neutrophil content groups.

### Construction of the WGCNA co-expression network

After grouping the TCGA-LUAD samples according to the characteristics of high or low neutrophil content, the gene expression data of TCGA-LUAD were analyzed with the “WGCNA” package^58^ to obtain the genes most related to neutrophil content. The samples were clustered to determine the overall correlation of all samples in the dataset and to exclude outliers (missing values and outliers). Correlation coefficient weighting was used to ensure that the connections between genes in the network followed a scale-free network distribution, and the soft threshold power β was selected based on the lowest power with a high value of the scale-free topological fitting index, with the minimum gene/module set to 100, to filter out highly similar modules for constructing the co-expression network. Finally, we conducted a correlation analysis between modules and traits to identify the modular genes most correlated with neutrophil content.

### Identification of neutrophil marker genes by single-cell sequencing analysis

We selected 11 primary LUAD samples from the single-cell dataset GSE131907 for subsequent analysis. We used the "Seurat" package^59,60^ to process 10× scRNA-seq data to build an S4 object and filter out low-quality cells according to the quality control standards: 300 < nFeature < 7500; 200 < nCount < 50,000; percent.mt < 10. First, we used the “NormalizedData” function to normalize the scRNA-seq data with the default normalization method and scaling factor. Then, we used the “FindVariableFeatures” function to identify the top 2000 highly variable genes and used the “ScaleData” function to normalize the scRNA-seq data. We performed PCA on the highly variable genes using the “RUNPCA” function to identify significant PCs. Based on the Euclidean distance of PCA, we used the “FindNeighbors” function, “FindClusters” function, and “RunTSNE” function for cell clustering analysis of the top 20 PCs, and then used the “FindAllMarkers” function to calculate the differentially expressed genes for each cluster. To identify marker genes for neutrophils, we used the “SingleR” package^61,62^, the CellMarker database (http://xteam.xbio.top/CellMarker/), and the PanglaoDB database (https://panglaodb.se/) to annotate cells in different clusters, and genes with log2FC absolute value > 1 and adjust *p*-value < 0.05 were considered as marker genes.

### Functional enrichment analysis of GO and KEGG

The obtained modular genes were intersected with the neutrophil marker genes acquired from analyses of scRNA-seq data to filter NRGs. We used the “clusterProfiler” package^63^ to perform the GO and KEGG functional enrichment analysis^64,65^ on the screened NRGs. GO analysis was performed using the “enrichGO” function, and GO annotation was based on the genome-wide annotation package published by the Bioconductor project (org.Hs.eg.db). KEGG analysis was performed using the “enrichKEGG” function. Adjust *p*-value < 0.05 was considered significantly enriched.

### Construction and validation of a prognostic model based on NRGs

We used the “survival” package to perform univariate COX regression analysis to evaluate the prognostic value of NRGs on OS in TCGA-LUAD patients, with *p*-value < 0.05 considered to be associated with prognostic relevance. Next, the LASSO regression algorithm in the “glmnet” package was used to establish the penalty coefficient and selection variables, and the ten-fold cross-validation method was used to determine the penalty coefficient (λ) of the regression model. Finally, based on the Lasso regression analysis of the selected optimal number of variables, the multivariate Cox regression analysis was further carried out with the “Coxph” function and the “step” function in R software. We calculated the risk score for each LUAD patient in the TCGA cohort according to the following formula:

$$risk \, score={\sum }_{i=1}^{n}\left[coefficient\left(genei\right)*expression\left(genei\right)\right]$$

The formula was determined from the linear combination of gene expression levels and weighted with the corresponding regression coefficients from the stepwise multivariate Cox proportional risk regression model. Based on the median cut-off values of the risk scores, we divided the TCGA-LUAD patients into high- and low-risk groups. Kaplan–Meier method and log-rank tests were used to analyze and compare the statistical significance of OS and survival differences between different risk groups. The result was considered to be statistically significant when *p*-value < 0.05. Time-dependent ROC curves were then plotted at 1, 3, and 5 years using the “timeROC” package to assess the efficacy of the prognostic model. When the AUC is greater than 0.6, it is considered to have great prediction ability. In addition, we used the “pheatmap” package and the “ggplot2” package to plot risk curves, survival status maps, and genetic risk heat maps for LUAD patients based on different risk groups to explore the relationship between risk scores, patients’ survival, and prognostic gene expression. Finally, we used the univariate and multifactorial COX analyses to identify the prognostic significance of risk score and clinical characteristics. To validate the predictive power of the model, GSE72094 was used as the validation set and the model was externally validated using K-M survival analysis and AUC. Based on the prognostic signature and clinical characteristics of samples, the “rms” package (https://cran.r-project.org/web/packages/rms/index.html) was used to construct the nomogram. The performance of the nomogram was evaluated using calibration curves and 1-, 3-, and 5-year ROC curves.

### Correlation analysis of clinicopathological characteristics of prognostic models

To explore the correlation between risk scores and clinicopathological characteristics (age, gender, tumor stage), only TCGA-LUAD samples with complete clinical information were retained. We used the “complexheatmap” package to create heat maps of individual clinical characteristics between high and low-risk groups and performed correlation analyses. The “ggplot2” package and the “ggpubr” packages were used for graphic visualization. We then used the “timeROC” package to construct a ROC curve based on risk scores and clinicopathological characteristics to compare the predictive power for 1-, 3- and 5-year survival in LUAD patients. Finally, we grouped patients according to different clinicopathological characteristics to compare the difference in OS between high- and low-risk groups.

### Tumor microenvironment analysis and gene set enrichment analysis

We used the “ESTIMATE” package for tumor microenvironment analysis and quantified the data of LUAD transcriptome profiling by stromal cell score (Stromal Score) and immune cell score (Immune score). The sum of the Stromal Score and Immune score is equal to the tumor microenvironment score (ESITIMAT Score). The ESITIMAT Score can be used to estimate tumor purity, the lower the ESITIMAT Score, the higher the tumor purity. Then, we explored the relationship between risk score and Stromal Score, Immune score, ESITIMAT Score, and tumor purity to investigate the correlation between tumor heterogeneity and risk scores. The pathways and functions enrichment analysis for the mRNAs associated with high- or low-risk groups was carried out using c5.go.symbols.gmt and c2.cp.kegg.symbols.gmt as gene sets database at 1,000 random sample permutations by the “GSEA” function of the “clusterProfiler” package. The enrichment functions or pathways were statistically significant when the *p*-value < 0.05.

### Immune correlation analysis

The “GSVA” package^66^ and the “GSEABase” package were used to analyze the differences in immune-related functions between high- and low-risk groups, and we further compared the expression levels of immune checkpoint-related genes between high- and low-risk groups. In order to investigate the response of high- and low-risk groups to different immunotherapies, the TIDE scores were calculated separately for each sample on the TIDE website (http://TIDE.dfci.harvard.edu/^67^, and the IPS of each TCGA-LUAD sample was obtained from TCIA^68^ to predict the sensitivity of different risk groups to CTLA-4 and PD-1 blockers.

### Drug screening and sensitivity analysis

We hope to further identify new potential targets and more effective drugs for the treatment of LUAD, and conduct drug screening and sensitivity analysis. The “oncoPredict” package was used to predict the therapeutic response of common targeted drugs and chemotherapeutic agents in cancer patients^69^. The package matches the gene expression profile to the half-maximal inhibitory concentration (IC50) of tumor cell lines to drugs, which comes from Genomics of Drug Sensitivity in Cancer (GDSC, https://www.cancerrxgene.org/) and Cancer Cell Line Encyclopedia (CCLE, https://sites.broadinstitute.org/ccle/). Non-paired t-test was used to analyze the drug sensitivity between the two groups. The *p*-value < 0.01 was considered to be statistically significant.

### Tissue samples collection and quantitative real-time PCR (qRT-PCR)

15 primary tumor tissues and adjacent normal tissues were collected from the LUAD patients in the Third Xiangya Hospital of Central South University, and informed consent was obtained from all patients. None of the patients had undergone chemotherapy, radiotherapy, target therapy or immunotherapy. The study was approved by the Medical Ethics Committee of the Third Xiangya Hospital of Central South University. Total RNA from tissues was extracted using Trizol reagent (Invitrogen, USA) following the manufacturer’s instructions. cDNA was synthesized using a reverse transcription kit (Accurate Biology, Hunan, China), qRT-PCR was performed using SYBR Green premix qPCR Kit (Accurate Biology, Hunan, China) on Roche LightCycler 480 II (Roche, Basel, Switzerland). ACTB was used as internal controls for the normalization. Relative mRNA expression levels were calculated using the 2−ΔΔCt method. The primer sequences are shown in Supplementary Table S2.

### Statistical methods

All statistical analyses were carried out by R software (version 4.2.1). Wilcoxon test and Kruskal–Wallis test were used to compare the two groups and more groups. Kaplan–Meier method was used to draw a prognostic survival curve, and a Log-rank test was used to evaluate the significance of statistical differences. The Spearman test was used for correlation analysis and correlation coefficient calculation.

### Institutional review board statement

The study was conducted in accordance with the Decla-ration of Helsinki, and approved by the Institutional Review Board of Third Xiangya Hospital OF Central South University (Fast 23438, 2023.07.03) for studies involving humans. Exemption from informed consent of all subjects involved in the study, subject to review and approval by the In-stitutional Review Board of Third Xiangya Hospital of Central South University.

### Informed consent statement

Exemption from informed consent of all subjects involved in the study, subject to review and approval by the Institutional Review Board of Third Xiangya Hospital of Central South University.

### Supplementary Information

Supplementary Information.

## Data Availability

The data presented in this study are openly available in the TCGA and GEO databases. The names of the repository/repositories and accession number(s) can be found below: https://portal.gdc.cancer.gov/, TCGA.GDC; https://www.ncbi.nlm.nih.gov/geo/query/acc.cgi?acc=GSE131907, GSE131907; https://www.ncbi.nlm.nih.gov/geo/query/acc.cgi?acc=GSE131907,GSE72094.
