# Supplementary material for: Construction and validation of a novel prognostic model of neutrophil‑related genes signature of lung adenocarcinoma
Source: Sci Rep. 2023 Oct 25;13:18226. doi: 10.1038/s41598-023-45289-8 (PMC10600204; doi:10.1038/s41598-023-45289-8)
Supplement: Supplementary file 1 — Supplementary Information. [file 41598_2023_45289_MOESM1_ESM.pdf]

Supplementary Material

Construction and validation of a novel prognostic model of neutrophil-related genes signature of lung adenocarcinoma

Qianjun Zhu[1,†], Yanfei Chai[1,2,†], Longyu Jin[1], Yuchao Ma[1], Hongwei Lu[2],2, Yingji Chen[1], Wei Feng[1*]

[1]Department of Cardiothoracic Surgery, Xiangya Third Hospital, Central South University, Changsha 410013, Hunan, China

[2]Center for Experimental Medicine, Third Xiangya Hospital, Central South University, Changsha, China

[*]Correspondence: Wei Feng, fweimail@163.com;

†These authors have contributed equally to this work and share the first authorship.

1 Supplementary Figures and Tables

1.1 Supplementary Figures

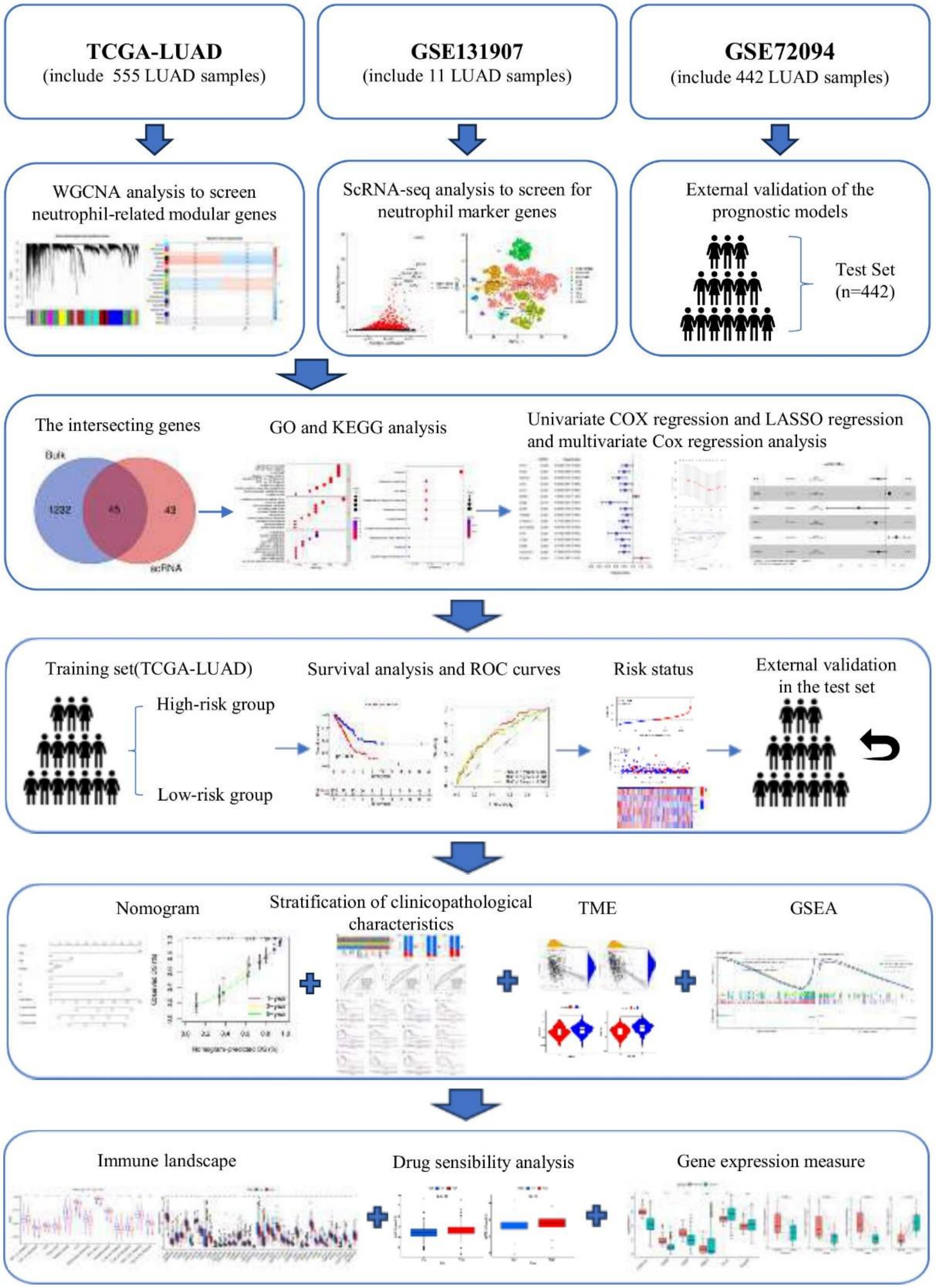
TCGA-LUAD
(include 555 LUAD samples)
GSE131907
(include 11 LUAD samples)
GSE72094
(include 442 LUAD samples)
WGCNA analysis to screen neutrophil-related modular genes
ScRNA-seq analysis to screen for neutrophil marker genes
External validation of the prognostic models
Test Set
(n=442)
The intersecting genes
Bulk
1232
45
43
scRNA
GO and KEGG analysis
Univariate COX regression and LASSO regression and multivariate Cox regression analysis
Training set(TCGA-LUAD)
High-risk group
Low-risk group
Survival analysis and ROC curves
Risk status
External validation in the test set
Nomogram
Stratification of clinicopathological characteristics
TME
GSEA
Immune landscape
Drug sensibility analysis
Gene expression measure

Supplementary Figure 1. Flowchart of this study.

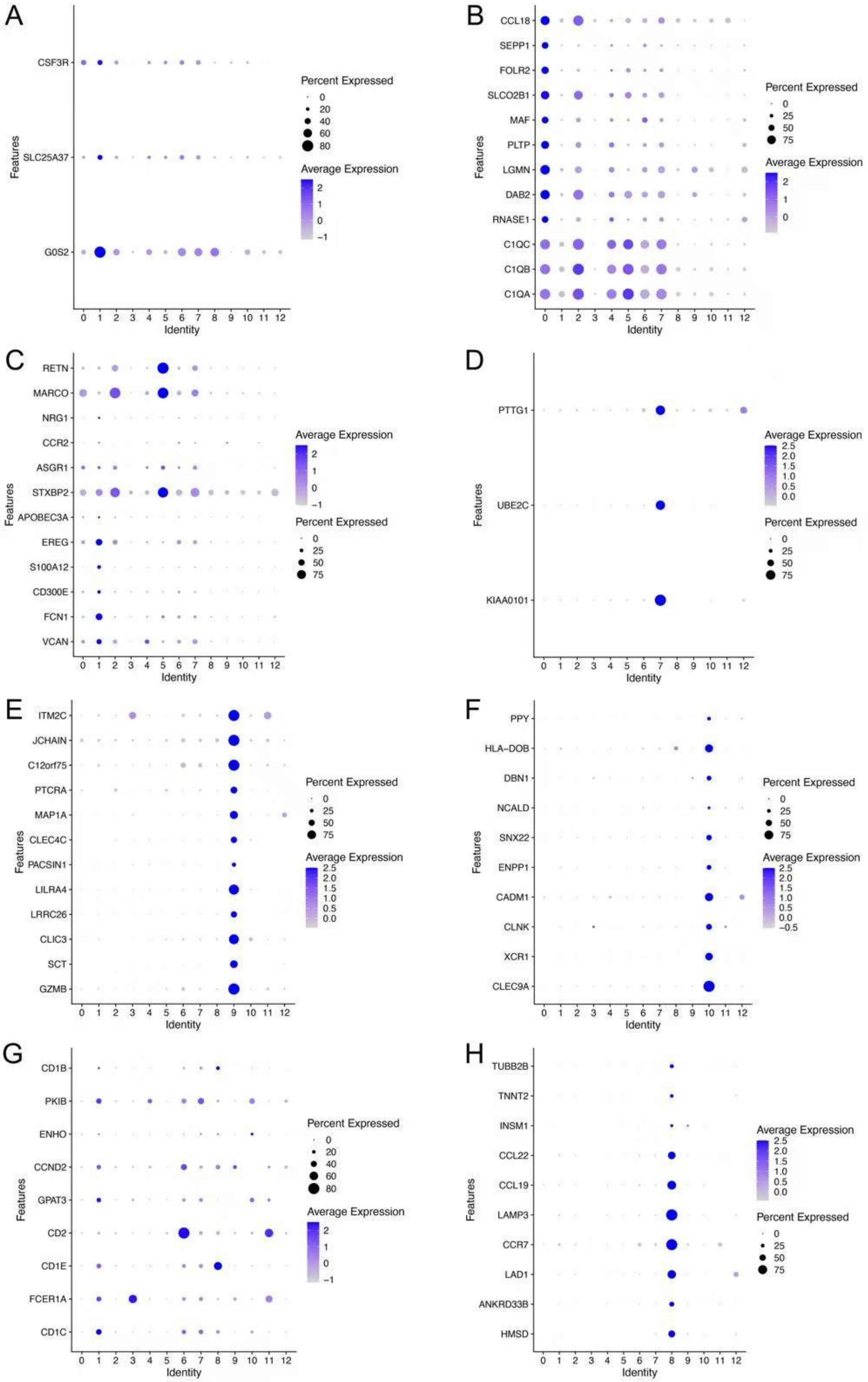

A
CSF3R
SLC25A37
G0S2
Features
Identity
0 1 2 3 4 5 6 7 8 9 10 11 12
Percent Expressed
0
20
40
60
80
Average Expression
2
1
0
−1
B
CCL18
SEPP1
FOLR2
SLCO2B1
MAF
PLTP
LGMN
DAB2
RNASE1
C1QC
C1QB
C1QA
Percent Expressed
0
25
50
75
Average Expression
2
1
0
C
RETN
MARCO
NRG1
CCR2
ASGR1
STXBP2
APOBEC3A
EREG
S100A12
CD300E
FCN1
VCAN
D
PTTG1
UBE2C
KIAA0101
2.5
2.0
1.5
1.0
0.5
0.0
E
ITM2C
JCHAIN
C12orf75
PTCRA
MAP1A
CLEC4C
PACSIN1
LILRA4
LRRC26
CLIC3
SCT
GZMB
F
PPY
HLA−DOB
DBN1
NCALD
SNX22
ENPP1
CADM1
CLNK
XCR1
CLEC9A
−0.5
G
CD1B
PKIB
ENHO
CCND2
GPAT3
CD2
CD1E
FCER1A
CD1C
H
TUBB2B
TNNT2
INSM1
CCL22
CCL19
LAMP3
CCR7
LAD1
ANKRD33B
HMSD

Supplementary Figure 2. The bubble maps of genes on the annotation of myeloid immune cell subpopulations. (A) Neutrophil, (B) Macrophage, (C) Monocyte, (D) GMP, (E) PDC, (F) DC1, (G) DC2, (H) DC3.

1.2 Supplementary Tables

Supplementary Table S1. The clinical baseline characteristics table of TCGA-LUAD and GSE72094 cohort in this study.

Characteristics	TCGA-LUAD(Training set)	GSE72094(Test set)
Total	486	442
Age		
≤65	227(46.7%)	127(28.7%)
> 65	240(49.4%)	294(66.5%)
Unknown	19(3.9%)	21(4.8%)
Gender		
Male	222(45.7%)	202(45.7%)
Female	264(54.3%)	240(54.3%)
Tumor Stage		
Stage I	262(53.9%)	265(60.0%)
Stage II	112(23.1%)	69(15.6%)
Stage III	79(16.3%)	63(14.3%)
Stage VI	25(5.1%)	17(3.8%)
Unknown	8(1.6%)	28(6.3%)
T stage		
T1	163(33.5%)	-
T2	260(53.5%)	-
T3	41(8.5%)	-
T4	19(3.9%)	-
Unknown/TX	3(0.6%)	-
N stage		
N0	312(64.2%)	-
N1	90(18.5%)	-
N2	70(14.4%)	-
N3	2(0.4%)	-
Unknown/NX	12(2.5%)	-
M stage		
M0	333(68.5%)	-
M1	24(4.9%)	-
Unknown/MX	129(26.6%)	-
Survival State		
Alive	304(62.6%)	298(67.4%)
Dead	182(37.4%)	122(27.6%)
Unknown	-	22(5.0%)

Supplementary Table S2. The primers sequences used in this study.

	Primers sequence (5′-3′)	
	Forward primer	Reverse primer
CYP27A1	ACCCAGTTTGTGTTCTGCCA	AAATGGGTGCTGGATCCTGG
CD69	TTTGCATCCGGAGAGTGGAC	CAGCACACAGGACAGGAACT
CD68	CACAGTCCTGCCACCACTAG	TGGGCAGAACTGGTGAATCC
PLTP	TCCAGCATGACTATGGACGC	AGAGGGGCCTGTAATGGGAT
ACTB	TCGTGCGTGACATTAAGGAG	TTGCCAATGGTGATGACCTG

Supplementary Table S3. The 1277 genes from the green module of the WGCNA analysis.

FGR	*CFH*	*LAP3*	*M6PR*	*ICA1*	*DBNDD1*	*CASP10*	*TFPI*
SLC7A2	*SKAP2*	*MCUB*	*PRKAR2B*	*MPO*	*LAMP2*	*CRLF1*	*CX3CL1*
TTC22	*GGCT*	*CEACAM21*	*NOS2*	*MATK*	*CAMK1G*	*IFFO1*	*STAB1*
IDS	*CD4*	*BTK*	*SYT7*	*PLAUR*	*TYROBP*	*ALOX5*	*MAMLD1*
WAS	*SLC11A1*	*ZNF582*	*MARCO*	*CYP24A1*	*CD74*	*CPS1*	*RRAGD*
NR1H3	*FAS*	*CD44*	*IFNGR1*	*TNFRSF1B*	*HMGB3*	*PNPLA6*	*HOXC8*
MSR1	*CDH10*	*LCP2*	*XK*	*VAMP3*	*EPN3*	*HEXB*	*MCUR1*
FSTL4	*RELT*	*FAM114A2*	*GYG2*	*DCBLD2*	*F7*	*TMCC3*	*TBXAS1*
ALDH18A1	*SLC2A3*	*BCAT1*	*ATP2C2*	*SNX24*	*CYB5R4*	*GSTO2*	*TBC1D1*
CD84	*SPI1*	*KLF6*	*KCNAB2*	*NUCB2*	*DAPP1*	*WIPI1*	*CAMK2A*
VASH1	*MYO3B*	*LNX1*	*ALDH3A2*	*P4HA2*	*FCGR2B*	*STK10*	*PANX2*
NTN4	*SCARF1*	*IPCEF1*	*KCNQ2*	*PAK3*	*APBB1IP*	*CAPZB*	*GPR137B*
BRINP1	*BPIFB2*	*SLC1A3*	*LXN*	*EPB41L2*	*OSTM1*	*ARG2*	*PKP1*
STK17B	*ATP8B1*	*EPB41L3*	*RNF13*	*HAL*	*CD82*	*FCN1*	*PILRA*
AKR1B1	*TTC39A*	*EIF2AK1*	*SNX10*	*LAT2*	*HSD17B14*	*LPCAT2*	*PIR*
SLC15A1	*SIGLEC1*	*P2RX7*	*CDIP1*	*RGS1*	*ICAM1*	*IRAK3*	*LYZ*
THPO	*CD209*	*MCOLN1*	*EFNB1*	*NLRC4*	*FH*	*CEBPE*	*TBX15*
ECHDC1	*UPRT*	*PTGS1*	*TLL2*	*TREM2*	*MYO9B*	*AMELY*	*OSM*
CYTH4	*MFNG*	*CRYBB1*	*HMOX1*	*APOL4*	*PVALB*	*KIAA0930*	*NCF4*
CSF2RB	*KCTD17*	*RIN3*	*LGMN*	*SUSD6*	*PLTP*	*MMP9*	*CD40*
CTSZ	*BIRC7*	*SIRPB1*	*HCK*	*SAMHD1*	*BPI*	*VAPA*	*VSIG1*
ATG4A	*STS*	*TLR8*	*MOSPD1*	*PLS3*	*RENBP*	*KLHL4*	*ACP5*
PLA2G15	*COTL1*	*MEFV*	*AQP8*	*CPPED1*	*AQP9*	*LACTB*	*EHD4*
OCA2	*RHOV*	*IL7*	*GSR*	*FGL1*	*MAN2B1*	*LYL1*	*RETN*
IL4I1	*LILRB1*	*LILRA1*	*IL27RA*	*CLC*	*TGFB1*	*CEACAM4*	*CD33*
SIGLEC6	*PLA2G4C*	*SIGLEC5*	*PLEKHA4*	*LILRB5*	*SLC5A5*	*TMEM59L*	*HAMP*
GSDME	*ATP6V0A4*	*TFEC*	*MET*	*VIPR2*	*CPED1*	*EVX1*	*CPVL*
CCL24	*GIMAP2*	*TRIM14*	*AMBP*	*TNFSF8*	*DDX58*	*BAG1*	*RASSF4*
PKD2L1	*VSIR*	*LIPA*	*SFXN3*	*KAZALD1*	*DNAJC12*	*TRIM16L*	*ENO3*
RASD1	*ALDH3A1*	*ICAM2*	*CCL7*	*CCL2*	*CCL8*	*CCL1*	*ABI3*
DUSP3	*SLC16A6*	*PHOX2B*	*OCIAD1*	*AREG*	*SLC2A9*	*BST1*	*UGDH*
PPARGC1A	*CTSC*	*MS4A6A*	*MS4A4A*	*FOLR3*	*APOA4*	*SLC15A3*	*HPS5*
CD69	*CLEC2B*	*SELPLG*	*CORO1C*	*SLC6A12*	*SH2B3*	*CREBL2*	*SCNN1A*
OAS2	*RASAL1*	*FZD10*	*TBC1D30*	*ACRBP*	*CLEC4A*	*DSE*	*GCNT2*
ADTRP	*MAN1A1*	*TPD52L1*	*CD83*	*CRYBG1*	*QKI*	*PDE10A*	*LY86*
HBEGF	*RNF130*	*ARSB*	*THBS4*	*ST8SIA4*	*TXNDC15*	*HRH2*	*HGD*
CD86	*OGG1*	*KAT2B*	*HES1*	*GNAI2*	*GNB4*	*PODXL2*	*CSPG5*
MAPKAPK3	*MOB1A*	*STEAP3*	*ITGA4*	*IFIH1*	*GCA*	*DOK1*	*EVA1A*
GLS	*IGFBP2*	*IL1R1*	*ID2*	*ODC1*	*PLEK*	*KISS1R*	*VAX2*
PAPPA2	*RAP1A*	*RNF19B*	*NCF2*	*PLA2G4A*	*GADD45A*	*RGS2*	*CTH*
WARS2	*GNPAT*	*RRAGC*	*KMO*	*PADI2*	*ACTL8*	*GBP3*	*RAB29*
CNN3	*F3*	*HSD11B1*	*IRF6*	*STX12*	*C1orf54*	*AKAP7*	*RAB32*
SGK1	*RARRES1*	*MFSD1*	*FKBP15*	*NEK6*	*NR4A3*	*CSF3R*	*FLVCR2*
ATL2	*EPCAM*	*OGFRL1*	*IFIT3*	*NKX2-3*	*IFIT2*	*MOB3B*	*INSL4*
CD274	*CXorf21*	*TNFSF18*	*TEX11*	*TGFBI*	*WASHC3*	*DUSP4*	*RDH10*
SCPEP1	*SPOP*	*LRAT*	*CSTA*	*CD80*	*FABP3*	*CCRL2*	*CCR2*
TNFSF10	*TMIGD3*	*PAEP*	*OBP2A*	*PRXL2A*	*WIPF3*	*GLIPR2*	*TAF1L*
SRGN	*EGR2*	*HVCN1*	*BHLHE41*	*ADGRE5*	*NCKAP1L*	*MMP19*	*PDE1B*
HOXC13	*HOXC11*	*IL13RA2*	*SLC36A1*	*BATF3*	*G0S2*	*RAP2C*	*RAB38*
INHA	*KCNK15*	*NAGK*	*IL17C*	*F13A1*	*MRS2*	*EEF1E1*	*EREG*
TMEM255A	*PTGER2*	*SLC25A19*	*SRMS*	*IL1B*	*INSIG2*	*ATG4C*	*DEFB126*
OVOL2	*BFSP1*	*LRRN4*	*BANF2*	*SIRPD*	*GDF5*	*ID1*	*GPR42*
HCST	*KRT36*	*STAT5A*	*TRAP1*	*CFP*	*EVI2A*	*ARMCX1*	*ZC4H2*
RGS13	*HIVEP3*	*ADGRE2*	*OR7C2*	*GNGT1*	*FGL2*	*YWHAH*	*TPST2*
RAC2	*APOBEC3A*	*IRF5*	*ARHGAP22*	*SOX15*	*CD68*	*KLK14*	*SIGLEC9*
KLK10	*KLK8*	*ARHGEF6*	*MAP7D3*	*NXNL2*	*MOSPD2*	*APOE*	*APOC1*
LRCH2	*LSP1*	*CALY*	*NPAS1*	*GMFG*	*THEMIS2*	*SLC6A8*	*MPP1*

LILRB2	BPIFA2	SH3BGRL	PPT1	ADGRE3	SH3BP5	PDLIM4	MGAT1
BARX1	KHDRBS3	PDHA1	TRIM21	PPARG	EMILIN2	ARFIP2	TRIM22
CARD6	CLEC10A	ATP1A4	BCAN	SYT11	SLC14A2	ALOX5AP	CHI3L1
MYBPH	CHIT1	PRAM1	LGALS12	PLAAT4	GIMAP6	TMEM254	AMPD3
IER3IP1	IRAK2	CAMK1	CHL1	GSTM1	PTPN22	CD101	RSAD2
MYCN	CMPK2	ANO3	TIMM17A	IL2RA	ACP2	SLC43A3	CLDN10
SLC37A2	OSTF1	ANXA1	CTSL	GOLM1	HAVCR2	SDS	OASL
TRAFD1	MDFIC	NT5E	PRR5L	STX11	KCNK1	GLUL	NPL
WNT10A	CYP27A1	PLXNC1	TBC1D4	RCBTB2	LCP1	GPNMB	IL6
AOAH	BZW2	MYO1G	TM6SF1	IL10	IL1RN	GYPC	KLF4
TMOD1	SLC31A1	TLR4	PIM1	CAPN11	KIAA0319	BPHL	TLR2
SLCO2B1	PRCP	SDCBP	DDX60	MMP7	ARHGAP20	TMPRSS13	CASP1
CASP5	TMEM62	SLC28A2	IFI44L	IFI44	SLC44A5	DNASE2B	CYP1B1
LOXL4	CH25H	TACC2	DUSP5	CALHM2	RBP4	CHRNA1	SLC40A1
APH1B	HERC6	HERC5	RASGEF1B	RAP1GDS1	NAAA	PAPSS1	SLC39A8
RGS3	PARVG	GLIPR1	MTMR6	GPR84	CELA1	ESYT1	LPAR6
RAB20	GRTP1	RTN1	GPR65	JDP2	SORD	LYSMD2	SLC27A2
ANP32A	BCL2A1	ARRDC4	PML	FURIN	ITGAX	IGSF6	MARVELD3
OSGIN1	IRF8	RPL3L	IMPA2	ARRB2	ZMYND15	ASGR1	PIK3R5
CARD14	TTYH2	SECTM1	MAPK4	TXNL4A	VAV1	HUNK	SIK1
TRPM2	EMP3	CBLC	MYO1F	NLRP12	SLC2A5	C1orf216	MOB3C
KCNA10	C1orf162	CD53	ATP1B1	FCGR2A	PRUNE1	ADAMTSL4	C1orf56
DUSP10	S100A8	CREB3L4	EFNA3	C1orf131	DEGS1	REN	RALB
CNGA3	CPNE9	STAC	IQSEC1	ARL6IP5	MYH15	ADPRH	NCEH1
SLC10A4	SNCA	DDIT4L	MARCHF1	LHFPL2	IQGAP2	TIMD4	TNIP1
KAAG1	PLA2G7	DOK3	PPP1R18	LGSN	FGD2	NFKBIE	ARHGAP18
TMEM140	SH3KBP1	MSN	CHST7	AWAT2	DOCK11	ATP6V1B2	DOK2
DNAJC5B	TRIM55	ATP6V0D2	DPYS	STOM	GBGT1	PROSER2	TMEM236
VAX1	FUOM	RGS10	PGAP2	SERPING1	C11orf49	MS4A3	OCSTAMP
FERMT3	TM7SF2	FXYD4	FCGR1A	LYPD6B	RGS18	IL18	PIP4K2A
RILPL2	SLC7A11	CACNA2D4	TMEM86A	NPAS3	KCTD14	FRMD4A	PTPRO
AKR1C2	INPP1	TDO2	CCL28	KCNE4	AP1S3	CYSLTR2	ARL11
ARL14EP	PSTPIP2	KCNK13	ZFP36L2	ANKRD22	IFIT5	HHEX	GGPS1
MR1	DAB2	BMP6	RASSF3	ADGRF1	ADPRHL1	CMTM7	JAZF1
CABYR	UBASH3B	PITPNC1	C10orf90	LY96	TMSB4Y	CXADR	VOPP1
KLF10	PI4K2A	SAMSN1	SLC7A7	PIK3AP1	VSIG4	CLIC2	MCU
KCNMA1	MAP3K7CL	TIAM1	TDRD9	SH3RF2	AIFM1	NTAN1	ODR4
NECAP2	TMED6	ST3GAL2	ETS2	MX1	SVOPL	CIB4	PRXL2C
MRAS	CNOT11	NCF1	TMED4	SLAMF8	NDUFS2	FCER1G	HOXB13
C1QC	ADPGK	ADIPOR1	M1AP	AGRP	ZYX	ABCG1	ICOSLG
ITGB2	FCN2	COX6B2	JAML	TLCD1	UBE2Q1	HK3	BDH1
SIGLEC11	ZNF385A	TREML1	CCDC42	CYB561A3	AKR7A3	LAPTM5	SDC3
NTNG1	NLRP3	PEA15	FCGR3B	ATF3	BPNT1	TFB2M	PKDCC
HAAO	PEX13	LRATD1	SLC16A14	CTSS	TNFAIP8L2	LYSMD1	CDC42EP3
ANTXR2	KBTBD8	EIF4E3	CXCR1	ARPC2	ADORA1	MNDA	IFI16
CD200R1	TIPARP	PTX3	ABHD6	PCOLCE2	MTMR14	PLB1	SLC6A20
CCR1	ARHGEF3	S100P	PGRMC2	CAMP	PPM1M	ETNPPL	PITX2
TLR3	FABP7	DCBLD1	DACT2	STK17A	BMT2	GPR85	HEY1
FABP5	CTSB	GPER1	DCSTAMP	SYK	TMEM71	CYBB	OTUD1
SPTSSA	FOLR2	SSX3	FAAH2	DRGX	NUDT5	TMEM52B	CASP7
LARGE2	KCNC2	SPRED1	GPT2	AMN	RAB8B	ASPG	TPP1
SERPINB8	WDR72	CLEC4D	SLC38A8	B2M	ANPEP	RBPMS2	MS4A6E
MS4A7	MS4A14	MAPRE2	PDIA3	B4GALNT2	GNGT2	BPIFB6	TBC1D2B
NOD2	DPEP2	RAB8A	TUBA1A	GPD1	LAIR1	KLK11	KLK13
CD300C	CD300A	GSDMA	CASKIN1	NAALADL1	RAB4A	PXK	MTNR1A
KLHL30	TXNDC2	CXXC4	MFF	LGALS9	SIGLEC7	AFAP1L2	P2RY12
GP2	RNASE2	RNASE3	PTAFR	RNASE6	CD52	HTRA4	PLEKHA2
GPR183	ZEB2	CHRNA5	SYAP1	ITGAM	ZPLD1	B3GNT2	KRT78
CD14	NUDT9	KRT83	SIX2	AC093323.1	OSCAR	PAQR8	CEACAM3
PKIA	FPR2	FPR1	ZDHHC16	CHST11	NLRP5	LRRC8C	PTGER4
FGG	FGA	FGB	PTCRA	P2RY6	GPR34	RGS19	TMEM51
SPATA5L1	RASGRP4	C3AR1	PRNP	KLF17	CYP4F11	MOB3A	SLFN12
AZU1	CLEC7A	CLEC12A	MANEA	MUCL1	SMPDL3A	SLFN11	MYD88
HPSE	HSPA6	CYSLTR1	ABLIM3	SYT12	PPP1R3B	C1QB	C1QA
OLR1	NLRP13	NMNAT1	PLK3	MARCHF3	NRROS	CBY2	TLR1
C11orf45	GALNTL6	CMKLR1	FAM241A	ADGRE1	P2RY14	CENPS	PPP2R2D
LRRC25	TSGA10IP	LIPT2	ETV4	NUPR1	GPX2	BNIP3	ENTHD1
HSD11B2	RNPEP	SLCO3A1	SHMT1	ERICH5	CD163	PGBD5	IL17RA
CD163L1	GRB2	GPR150	PRSS36	MAF	OTOS	KCTD12	CD300LB
RNF186	MRFAP1L1	ZFP42	HES7	EDC3	HLA-DQB1	EGR3	ALOX12B
SLC17A8	ZBTB42	LACC1	NLRP8	NLRP11	GPBAR1	TMEM150B	BHLHA15
MB21D2	HOXC9	HOXC10	CXCR2	GREM2	NQO1	RPH3AL	FRAT2
CCL13	RAP2B	P2RY13	YIPF6	MACIR	COPG1	SLC9A9	RELL1
UBE2E2	GABRG3	AP1S2	GLTPD2	SPNS3	CSF1R	MCEMP1	SLC8A1
RIMBP3C	CCBE1	TRIM61	MX2	ACSM5	TANGO2	ZNF438	FAM3B
SRARP	NPW	ADAP2	ADRA2C	MRGPRE	CSF1	SOCS3	LPAR5
LRRC26	APOBR	RBM43	TMEM106A	ADSS1	MFSD6L	IL3RA	TCN2

FAM174B	*STAC3*	*ROR1*	*PDE6G*	*KRT79*	*ZFP36L1*	*IFIT1*	*PIGP*
THNSL1	*FFAR3*	*IRS2*	*CD300LF*	*DEFB131A*	*FFAR4*	*BPIFB4*	*EDARADD*
MTARC1	*CD300E*	*GLDN*	*FCAR*	*KLK12*	*BCDIN3D*	*CYP27C1*	*LILRB4*
TMEM17	*KRTAP15-1*	*GPR141*	*MITF*	*LILRA5*	*AKR1C1*	*CHP1*	*FPR3*
KCNJ11	*TRPV2*	*CARD9*	*SOWAHD*	*RAB42*	*HYKK*	*PEAK3*	*PLSCR1*
COL25A1	*DPYD*	*PARVB*	*ENTPD8*	*HACD4*	*LITAF*	*VSTM1*	*NKAPL*
ALG1L	*TDRD7*	*HLA-DRB1*	*AKR1C3*	*SPATS2L*	*SIRPB2*	*PTPN1*	*TPK1*
TLR7	*HLA-DQA1*	*GM2A*	*CD47*	*CASP4*	*SIGLEC15*	*GAL3ST4*	*IL27*
ZNF165	*C5AR1*	*STMN3*	*SPN*	*SLC28A3*	*MYO5A*	*ATG7*	*MPEG1*
PDCD1LG2	*NMB*	*PSAP*	*HOXC6*	*CFD*	*MAP1LC3C*	*FAM118B*	*SGTB*
CYRIA	*HES5*	*MBP*	*FCGR1B*	*SIRPA*	*DEFB107B*	*CLEC4C*	*BPIFA1*
CSF2RA	*SLC29A3*	*HLA-DRB5*	*TUSC1*	*ABCA4*	*SMOC1*	*SUCNR1*	*NAGA*
TGM2	*CR1*	*RAET1G*	*FCGR3A*	*OR6K3*	*DDO*	*SAMD13*	*MAFB*
GGTA1	*TMEM273*	*NPY4R*	*GPRIN2*	*HLA-DOA*	*HLA-DMA*	*HLA-DRA*	*SP5*
CARD16	*AIF1*	*LST1*	*MICB*	*C6orf136*	*LILRB3*	*ZFP57*	*SPDYC*
LGALS7	*MUC12*	*SNX2*	*MT1M*	*SAMD9*	*CRLF2*	*CLEC6A*	*JPT2*
ATP10A	*SLC48A1*	*LEPROT*	*GPSM3*	*C19orf38*	*FOXI3*	*KCNU1*	*CKMT1A*
HLA-DPB1	*HNRNPA1P48*	*AL603764.2*	*TMEM233*	*C2CD4D*	*HSBP1L1*	*PRAC2*	*ACBD6*
HLA-DPA1	*TNF*	*PET117*	*DEFB130A*	*RAB6D*	*APOC2*	*NFAM1*	*CKMT1B*
HLA-DQA2	*TNFSF12*	*LILRA4*	*LILRA2*	*SMKR1*	*RDH14*	*MIF*	*PLEKHO2*
HLA-DMB	*GNG10*	*LEFTY1*	*LY6G6D*	*LILRA6*	*APOBEC3C*	*NAIP*	*CCDC71L*
NACA2	*LYN*	*SIGLEC14*	*SIGLEC12*	*CHMP1B*	*CTSO*	*MTRNR2L10*	*CLEC5A*
RNASE12	*ITGB3*	*MRC1*	*TPBGL*	*NPY4R2*	*RBM8A*	*S1PR2*	*TRABD2B*
MTRNR2L11	*LIX1L*	*MILR1*	*C2orf15*	*CCL23*	*CCL18*	*H4C7*	*RIMBP3*
SIK1B	*CCL3L3*	*PIK3R6*	*FP565260.3*	*MARCKS*	*CCL3*	*ADORA3*	*LMLN2*
TAF11L11	*SCYGR2*	*SCYGR7*	*AL031315.1*	*APOC4-APOC2*			

Supplementary Table S4. The Third levels of dimensionality reduction and clustering: myeloid immune cells.

Cluster	Cell Type	Marker Gene
C0, C4, C6	Macrophage	*C1QA, C1QB, C1QC, RNASE1, DAB2, LGMN, PLTP, MAF, SLCO2B1, FOLR2, SEPP1, CCL18*
C1	Neutrophil	*G0S2, SLC25A37, CSF3R*
C2, C5	Monocyte	*VCAN, FCN1, CD300E, S100A12, EREG, APOBEC3A, STXBP2, ASGR1, CCR2, NRG1, MARCO, RETN*
C3, C11	DC2	*CD1C, FCER1A, CD1E, CD2, GPAT3, CCND2, ENHO, PKIB, CD1B*
C7	GMP	*KIAA0101, UBE2C, PTTG1*
C8	DC3	*HMSD, ANKRD33B, LAD1, CCR7, LAMP3, CCL19, CCL22, INSM1, TNNT2, TUBB2B*
C9	PDC	*GZMB, SCT, CLIC3, LRRC26, LILRA4, PACSIN1, CLEC4C, MAP1A, PTCRA, C12orf75, JCHAIN, ITM2C*
C10	DC1	*CLEC9A, XCR1, CLNK, CADM1, ENPP1, SNX22, NCALD, DBN1, HLA−DOB, PPY*
C12	Unknown	-

Supplementary Table S5. The 88 neutrophil marker genes obtained from scRNA-seq data analysis.

G0S2	*IL1B*	*FCN1*	*S100A12*	*CD1C*	*LST1*	*CORO1A*	*SERPINB9*
COTL1	*GPNMB*	*CTSD*	*APOC1*	*APOE*	*NAMPT*	*NUPR1*	*FTL*
LGALS2	*GLUL*	*GPR183*	*PPIF*	*PLAUR*	*CSTB*	*CD1E*	*CFP*
CLEC10A	*CD63*	*INSIG1*	*CDKN1A*	*C1QB*	*GCHFR*	*C1QA*	*JUN*
ACP5	*NR4A3*	*PLD3*	*REL*	*EREG*	*BTG1*	*MSR1*	*CXCL8*
LGALS3	*C1QC*	*DAB2*	*IER3*	*CD59*	*CD68*	*CYP27A1*	*CD9*
MS4A4A	*LIPA*	*CTSB*	*MARCO*	*BCL2A1*	*TREM2*	*PLTP*	*ATP2B1*
SERPING1	*CD163*	*STMN1*	*SOD2*	*CTSL*	*TIMP1*	*AREG*	*CCL18*
HLA-DPB1	*FABP5*	*CTSZ*	*LGMN*	*C15orf48*	*IFI27*	*IL1RN*	*HMOX1*
RNASE1	*FN1*	*FCER1A*	*IFI6*	*LTB*	*SEPP1*	*VCAN*	*S100B*
HPGDS	*TPSB2*	*TWISTNB*	*FBP1*	*SPP1*	*CD69*	*CCL2*	*S100A8*

Supplementary Table S6. The 45 intersecting genes.

C1QC	*GPNMB*	*PLTP*	*APOE*	*FABP5*	*SERPING1*	*FCN1*	*NUPR1*

CTSB	*LIPA*	*MSR1*	*LST1*	*EREG*	*G0S2*	*AREG*	*NR4A3*
S100A8	*CCL2*	*CD68*	*BCL2A1*	*CD163*	*HMOX1*	*COTL1*	*DAB2*
CCL18	*ACP5*	*LGMN*	*GPR183*	*TREM2*	*MS4A4A*	*CYP27A1*	*HLA-DPB1*
GLUL	*CTSL*	*C1QB*	*IL1RN*	*IL1B*	*MARCO*	*C1QA*	*CFP*
CTSZ	*CD69*	*CLEC10A*	*PLAUR*	*APOC1*			

Supplementary Table S7. Six neutrophil-related genes of prognostic signature.

Gene	Coefficient	HR	HR.95L	HR.95H	*p*-value
PLTP	-0. 1489	0.8616	0.7395	1.0039	0.0561
EREG	0.0722	1.0749	0.9997	1.1557	0.0509
CD68	-0.5497	0.5771	0.3053	1.0909	0.0906
CD69	-0.1891	0.8277	0.6956	0.9850	0.0332
PLAUR	0.2204	1.2466	1.0685	1.4543	0.0051
CYP27A1	-0.1398	0.8695	0.7190	1.0516	0.1496

Supplementary Table S8. The list of abbreviations.

LUAD	Lung adenocarcinoma
NRGs	Neutrophil-related genes
WGCNA	weighted gene co-expression network analysis
scRNA-seq	single-cell RNA-sequencing
LASSO	Least absolute shrinkage and selection operator
TIDE	Tumor Immune Dysfunction and Exclusion
IPS	Immune cell Proportion Score
TME	Tumor microenvironment
SCLC	Small-cell lung carcinoma
NSCLC	Non-small-cell lung carcinoma
ECM	Extracellular Matrix
ROS	Reactive oxygen species
NETs	Neutrophil extracellular traps
TANs	Tumer-associated neutrophils
TCGA	The Cancer Genome Atlas Program
GEO	Gene Expression Omnibus
nFeature	The number of genes

nCount	The sequence count per
percent. mt	The percentage of mitochondrial genes
PCA	Principal component analysis
PCs	Principal Components
NK	Natural killer
DCs	Dendritic Cells
GMP	Granulocyte-macrophage progenitors
PDCs	Plasmacytoid dendritic cells
GO	Gene Ontology
BP	Biological Process
CC	Cellular Component
KEGG	Kyoto Encyclopedia of Genes and Genomes
OS	Overall Survival
ROC	Receiver Operating Characteristic
AUC	Area Under Curve
GSEA	Gene Set Enrichment Analysis
ssGSEA	single sample Gene Set Enrichment Analysis
ICB	Immune Checkpoint Blockade
PD-1	Programmed Death 1
CTLA4	Cytotoxic T Lymphocyte Antigen-4
TCIA	The Cancer Immune Atlas
TMB	Tumor Mutation Burden
MSI	Microsatellite Instability
ICIs	Immune Checkpoint Inhibitors
FPKM	Fragments Per Kilobase Per Million

IC50	The half-maximal inhibitory concentration
GDSC	Genomics of Drug Sensitivity in Cance
CCLE	Cancer Cell Line Encyclopedia
qRT-PCR	quantitative real-time PCR
